# Altered Steroidome in Women with Gestational Diabetes Mellitus: Focus on Neuroactive and Immunomodulatory Steroids from the 24th Week of Pregnancy to Labor

**DOI:** 10.3390/biom11121746

**Published:** 2021-11-23

**Authors:** Leona Ondřejíková, Antonín Pařízek, Patrik Šimják, Daniela Vejražková, Marta Velíková, Kateřina Anderlová, Michala Vosátková, Hana Krejčí, Michal Koucký, Radmila Kancheva, Michaela Dušková, Markéta Vaňková, Josef Bulant, Martin Hill

**Affiliations:** 1 Institute of Endocrinology, 116 94 Prague, Czech Republic; londrejikova@endo.cz (L.O.); dvejrazkova@endo.cz (D.V.); mvelikova@endo.cz (M.V.); mvosatkova@endo.cz (M.V.); rkanceva@endo.cz (R.K.); mduskova@endo.cz (M.D.); mvankova@endo.cz (M.V.); jbulant@endo.cz (J.B.); 2 Department of Gynecology and Obstetrics, First Faculty of Medicine, General University Hospital in Prague, Charles University in Prague, 128 08 Prague, Czech Republic; antonin.parizek@lf1.cuni.cz (A.P.); Patrik.simjak@lf1.cuni.cz (P.Š.); katerina.anderlova@lf1.cuni.cz (K.A.); hana.krejci@lf1.cuni.cz (H.K.); michal.koucky@lf1.cuni.cz (M.K.)

**Keywords:** gestational diabetes mellitus, steroidome, neuroactive steroids, immunoprotective steroids, gestational age, maternal blood, mixed cord blood, gas chromatography-tandem mass spectrometry

## Abstract

Gestational diabetes mellitus (GDM) is a complication in pregnancy, but studies focused on the steroidome in patients with GDM are not available in the public domain. This article evaluates the steroidome in GDM+ and GDM− women and its changes from 24 weeks (± of gestation) to labor. The study included GDM+ (*n* = 44) and GDM− women (*n* = 33), in weeks 24–28, 30–36 of gestation and at labor and mixed umbilical blood after delivery. Steroidomic data (101 steroids quantified by GC-MS/MS) support the concept that the increasing diabetogenic effects with the approaching term are associated with mounting progesterone levels. The GDM+ group showed lower levels of testosterone (due to reduced AKR1C3 activity), estradiol (due to a shift from the HSD17B1 towards HSD17B2 activity), 7-oxygenated androgens (competing with cortisone for HSD11B1 and shifting the balance from diabetogenic cortisol towards the inactive cortisone), reduced activities of SRD5As, and CYP17A1 in the hydroxylase but higher CYP17A1 activity in the lyase step. With the approaching term, the authors found rising activities of CYP3A7, AKR1C1, CYP17A1 in its hydroxylase step, but a decline in its lyase step, rising conjugation of neuroinhibitory and pregnancy-stabilizing steroids and weakening AKR1D1 activity.

## 1. Introduction

Gestational diabetes mellitus (GDM) is a serious pregnancy complication, in which women without previously diagnosed type 2 diabetes (DM2) develop persistent hyperglycemia during pregnancy. Hyperglycemia during pregnancy is generally induced by glucose intolerance due to the dysfunction of pancreatic β-cells in context with chronic insulin resistance (IR). Advanced maternal age, obesity, and a family history of diabetes are common risk factors of GDM. GDM is regularly associated with macrosomia, birth complications of the infant, increased risk of maternal cardiovascular disease, and predisposition to DM2 in both mother and child [1]. The prevalence of GDM is about 15–20% of pregnancies worldwide, with an increasing trend mostly linked to accelerating obesity [1]. A number of interrelated factors (including steroids) affecting both insulin secretion (IS) and IR are involved in GDM pathophysiology [1].

The normal pregnancy is accompanied by higher IR, reduced hepatic insulin extraction and glucose effectiveness, higher levels of leptin, lower adiponectin concentrations, increased lipolysis and higher levels of triglycerides [2,3]. The pregnancy complicated with GDM is additionally associated with increased levels of inflammatory cytokines, higher macrophage infiltration and increased lipolysis. In contrast to healthy pregnancy with mild IR, the pregnancy complicated with GDM exhibits severe IR, hyperglycemia, leptin resistance, hyperlipidemia and oxidative stress. Women with GDM are unable to compensate for the IR of pregnancy by intensified IS [4].

In late gestation, the maternal system switches from the anabolic to the catabolic phase. Despite this transformation, maternal blood glucose remains constant or even slightly declines with advancing gestation, primarily due to the increased IS [4]. However, in mothers with GDM, the β-cells may deteriorate due to excessive insulin production persistently exhausting the cells. Furthermore, GDM is also associated with upregulated gluconeogenesis in the liver. While in normal pregnancy the maternal insulin sensitivity returns to pre-pregnancy levels shortly after labor, in GDM patients the disbalance frequently persists in the postpartum period and may develop to overt DM2 [1].

PregS and DHEAS stimulate IS through positive modulation of non-selective type 3 melastatin ion channels (TRPM3) [5,6]. Furthermore, PregS activates signal cascades involved in the amplified expression of activator protein 1 (AP-1) in the β-cells. In addition to TRPM3, L-type Ca^2+^ channels are also involved in β-cell activation [7]. PregS may also be involved in IS through positive modulation type 1 melastatin ion channels (TRPM1) [8]. Besides PregS and another abundant steroid, conjugate isopregnanolone sulfate reaches micromolar concentrations during pregnancy and inhibits specific proton-activated outwardly rectifying anion channels (PAORAC), which may further contribute to the increased β-cell activity [9].

However, the effects of PregS on IS do not appear to be unidirectional. PregS inhibits nicotine-acetylcholine receptors (nAChR) and restrains the influx of Ca^2+^ and Na^+^ ions into β-cells, thereby suppressing IS [10] and DHEAS regardless of pregnancy status, has a similar effect [11].

An increased activity of β-cells in the third trimester of gestation is linked to an increase in progesterone levels. It is an adaptive response to the increasing energy requirements of a fetus requiring increasing glucose and lipid intake. Progesterone is known as a diabetogenic steroid that acts through suppression of glucose transporter type 4 expression (GLUT-4) [12]. Unlike prolactin and hPL, which stimulate IS, progesterone suppresses their effects by down-regulation of β-cell function in the later stages of gestation [12]. Also, nuclear progesterone receptors may be involved in GDM pathophysiology [13]. These receptors are expressed in the pancreas, predominantly in glucagon-producing cells [14]. Unlike sulfated Δ^5^ steroids, progesterone inhibits the permeability of TRPM3 channels at levels typical for the luteal menstrual phase [15].

Alternatively, progesterone at concentrations common for the luteal menstrual phase and DHEA activate purinergic ionotropic P2X receptors [16]. The subtypes of P2X receptors are expressed in different tissues, including β-cells, and facilitate the influx of Ca^2+^ and Na^+^ ions at low glucose concentrations, followed by the depolarization of the cell membrane and IS [17].

While PregS and allopregnanolone inhibit the activity of AMPA/kainate receptors [18,19] and hence glucagon secretion, estradiol may function as their antagonist [20]. The β-cells produce significant amounts of γ-aminobutyric acid (GABA), which activates type A GABA receptors (GABA_A_R) and subsequently inhibits glucagon secretion in pancreatic α-cells [21]. At low concentrations, GABA increases IS, but inhibits it at higher concentrations [22]. GABAergic progesterone metabolites, such as allopregnanolone and pregnanolone in concentrations common for fetal and maternal circulation, increase GABA_A_R permeability for chloride ions [23,24], while sulfated Δ^5^ steroids are their antagonists [23,25]. The effect of listed GABAergic steroids on IS can therefore be considered as well.

Estradiol levels in the maternal circulation exceed by roughly two orders of magnitude the values common in the luteal phase of the menstrual cycle [26]. Estrogens promote energy consumption and food intake at the central level, increase glucose uptake in muscles and adipocytes, and reduce liver gluconeogenesis [27]. Estradiol binds to estrogen receptors (ER), thus modulating the transcription of target pancreatic genes [12]. Type α ER (ERα) are included in IS and nutrient homeostasis and type β ER (ERβ) augments IS. Furthermore, G protein-coupled ER (GPER), is also involved in IS [27]. ERα, which are expressed in pancreatic islets [28], stimulate glucose transporter 4 (GLUT4) expression, which is a limiting step in insulin-induced glucose uptake in skeletal muscles [12]. In addition, ERα may also operate at the central level, as disruption of ERα leads to increased visceral adiposity, hyperphagia, hyperglycemia, and impaired energy exposure [29].

In pancreatic β-cells, estradiol increases cyclic guanosine monophosphate levels and protein kinase G activity, which subsequently reduces K_ATP_ activity through the rapid and reversible closure of K_ATP_ channels. In synergy with increased glucose levels, estradiol depolarizes the cell membrane, triggering electrical activity and Ca^2+^ influx into the cell via activation of L-type Ca^2+^ channels. In addition, estradiol inhibits the permeability of voltage dependent potassium channels, which also activates the β-cells [12,30]. It should be mentioned that the lack of estradiol leads to a decrease of ATP-sensitive potassium channels (K_ATP_) gene expression and a consequent decrease in IS [12].

Low glucose levels induce increased permeability of AMPA/kainate receptors for Ca^2+^ ions and the opening of voltage dependent Ca^2+^ channels, which stimulates glucagon secretion [31]. In glucagon synthesizing α-cells, estradiol suppresses Ca^2+^ oscillations generated by low glucose levels and thus reduces glucagon secretion. [32]. Lastly, the estrogens increase the CNS and serum levels of GABAergic allopregnanolone [33].

Data from the literature indicate that the effect of steroids on glucose homeostasis is complex, and their resulting effect is likely to be contingent upon a variety of incremental effects that may be mutually antagonistic. Furthermore, while a short-term increase in the levels of some steroids may promote the IS, the long-term hyperstimulation of the β-cells during pregnancy may result in impaired IS due to exhaustion of the β-cells, which could be related to GDM pathophysiology [34]. In addition, it is also common in neuroactive steroids for the same substances to be neuro-excitatory on some receptors affecting glucose homeostasis, while acting as neuro-inhibitors on others. There is also a significant amount of data explaining the association between cortisol synthesis and its metabolism on one hand and disturbed glucose homeostasis on the other [35], yet less information is available about the anti-glucocorticoid and anti-inflammatory effects of 7-oxygenated androstanes in this respect [36,37], and particularly if related to the pathophysiology of GDM [38].

Although current evidence indicates a significant role for steroids in the development of GDM and in spite of the fact that steroid levels in pregnancy usually reach values 1–3 orders of magnitude higher compared to the situation in non-pregnant subjects, the mechanism of the influence of individual risk factors on the onset and course of GDM is not yet fully understood. The data regarding the role of steroids in the pathophysiology of GDM is deficient, and a study focused on alterations of the steroidome in women with GDM is lacking. The aim of this study was to evaluate the alterations in the maternal and fetal steroidome in GDM+ women and GDM− women from week 24 of pregnancy to birth and their relations to the physiology of pregnancy and parturition and to the pathophysiology of GDM.

## 2. Materials and Methods

### 2.1. Subjects, Sample Collection, Serum Preparation, and Storage

The study population consisted of two groups. The GDM+ group included pregnant women diagnosed with gestational diabetes via a 75 g, 2-h oral glucose tolerance test using IADPSG criteria (*n* = 44, 21 female newborns, 23 male newborns). The GDM− group involved controls without GDM (*n* = 33, 21 female newborns, 12 male newborns). The following three samples were collected from all participants: week 24–28 of gestation (collection of blood from maternal cubital vein), week 30–36 of gestation (collection of blood from maternal cubital vein), labor (collection of blood from maternal cubital vein before delivery). The fourth sample was collected from the mixed umbilical cord blood after delivery. Serum was obtained from blood sample after centrifugation for 5 min at 2000× *g* at 0 °C and stored at −20 °C until analyzed.

The study was conducted according to the Declaration of Helsinki, the International Conference of Harmonization (ICH) principles of Good Clinical Practice (GCP), and approved by the Ethics Committee of the Institute of Endocrinology (16 May 2016). After obtaining Ethics Committee approval, written informed consent was obtained from all patients. They were given sufficient time to discuss the study with their physician, to raise questions and to decide whether to participate or not. After fulfilling the inclusion study criteria, the participants were enrolled in the study.

### 2.2. Analytical Method

In total, 101 analytes were quantified in the blood from the cubital vein and in mixed umbilical cord blood of women with GDM and in corresponding controls. The steroids were measured using our previously published GC-MS/MS method [39]. The analytes, which were significantly related to GDM, are shown in Table 1, while those unrelated to GDM are displayed in Table S1.

### 2.3. Terminology of Steroid Polar Conjugates

The term steroid sulfate was used in the case of the dominance of 3α/β-monosulfate over other forms of steroid conjugates, while the term conjugated steroid was used in the case of comparable amounts of conjugate forms (sulfates, disulfates, and glucuronides). This terminology was based on the relevant literature, with appropriate citations for each steroid as follows: pregnenolone sulfate (PregS) [40,41], 20α-dihydropregnenolone sulfate, dehydroepiandrosterone sulfate (DHEAS) [41,42,43], 16α-hydroxy-DHEA sulfate [41,44], androstenediol sulfate [41,42], allopregnanolone sulfate [45], isopregnanolone sulfate [46], conjugated pregnanolone (sulfate + glucuronide [45]), 5α-pregnane-3β,20α-diol sulfate (3β,20α-disulfate + 3β-sulfate) [45], conjugated 5β-pregnane-3α,20α-diol (3β,20α-disulfate + 3β-sulfate + glucuronide) [45], androsterone sulfate [41,42], epiandrosterone sulfate [41,42], etiocholanolone sulfate [44], epietiocholanolone sulfate, conjugated 5α-androstane-3α,17β-diol (sulfate + glucuronide [42]), and conjugated 5α-androstane-3β,17β-diol (sulfate + glucuronide [42]).

### 2.4. Statistical Analysis

The data processing for steroid levels in the samples of mixed umbilical blood at labor was carried out using an ANOVA model consisting of factors GDM (+/−), Gender (of the newborn) (F/M), and Thyropathy (+/−) (in mother). The factors Gender and Thyropathy were included for adjustment as levels of some steroids depended on these factors and their effect should be separated from the effect of GDM. The evaluation of steroid levels in the samples of blood from the maternal cubital vein in three stages of the study was completed using an ANOVA model consisting of factors Subject explaining the inter-individual variability, between-subject factor GDM (+/−), within-subject factor Stage (of the study) (week 24–28 of gestation, week 30–36 of gestation, labor), interaction GDM × Stage, and between-subject factors for the adjustment Gender (F/M) and Thyropathy (+/−). The ANOVA testing was followed by Bonferroni multiple comparisons. The level of statistical significance was set to *p* < 0.05.

Respecting the skewed data distribution and non-constant variance in most dependent variables, these were transformed by power transformations to achieve data symmetry and homoscedasticity prior to further data processing [47]. The homogeneity and distribution of the transformed data were checked by residual analysis as described elsewhere [48]. The statistical software Statgraphics Centurion 18 Version 18.1.06 from Statgraphics Technologies, Inc. (The Plains, VA, USA) was used for the statistical analysis. Due to the absence of preliminary data for 101 steroids and limited sample size, the power analysis based on the F-test was completed post hoc. In the case of significant factor GDM and/or significant GDM × Stage interaction, the well-powered analyses (WP) and underpowered analyses (UP) were added in corresponding tables. For the WP analyses, the *p*-value was set to 0.05 and power to 0.8. The power analysis was performed using PASS 16 Power Analysis and Sample Size Software (2018) from NCSS, LLC. (Kaysville, UT, USA).

## 3. Results

### 3.1. GDM and Steroidome in the Mixed Umbilical Blood after Delivery of Placenta and Fetus

**Figure 1:**
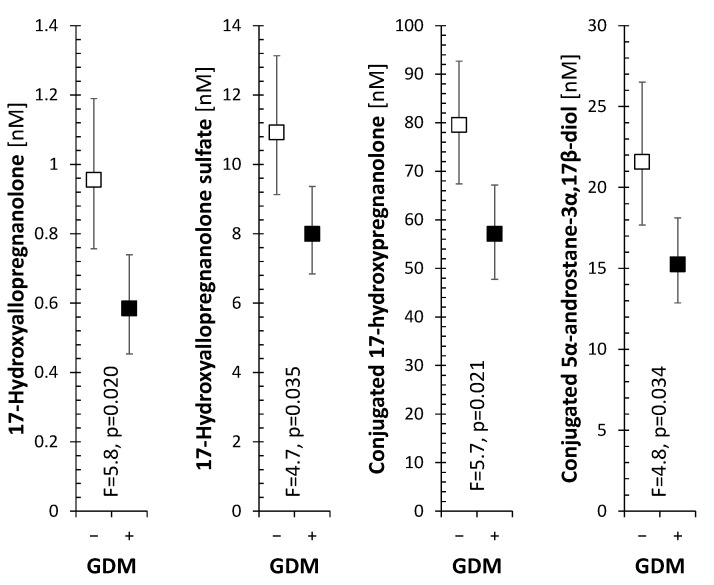
Levels of steroids showing significant differences between GDM− and GDM+ groups in mixed umbilical cord blood as evaluated by ANOVA adjusted to sex of the fetus and presence of thyropathy in mother. Empty and full squares with error bars represent retransformed means with their 95% confidence intervals for GDM− and GDM+ women, respectively. F is the F-statistics and *p* is the *p*-value for the factor GDM.

Only four significant GDM−related steroid changes were found in the mixed umbilical cord blood, such as lower levels of 5α/β- and 20-oxo/20α-reduced metabolites of 17 hydroxyprogesterone and conjugated 5α-androstane-3α,17α-diol indicating lower activity of the CYP17A1, particularly in the 17-hydroxylase step (see Figure 1 and Table S2).

### 3.2. Alterations in Steroidome in the Maternal Blood Related to GDM

A number of significant steroidomic changes were found in mothers with GDM (GDM+), indicating the primary role of the maternal compartment in GDM pathophysiology in terms of steroidomics. Out of the total 101 steroids in GDM+ mothers, six showed higher levels, 41 steroids had lower levels, two steroids showed insignificant factor GDM but significant interaction GDM × Stage (Table 1). Fifty-two steroids were unrelated to GDM (Table S1). The levels of all steroids under investigation in the maternal venous blood for GDM+ and GDM− women in three stages of the study (week 24–28 of gestation, week 30–36 of gestation, Labor) are shown in Table 1 and Table S1.

#### 3.2.1. Δ^5^ Steroids

From the Δ^5^ pregnane steroids (8 analytes), the levels of two of them (pregnenolone and 20α-dihydropregnenolone sulfate) were higher in GDM+ women, the levels of 16α-hydroxypregnenolone sulfate were lower in GDM+ women and the levels of five steroids pregnenolone sulfate, 17-hydroxypregnenolone, 17-hydroxypregnenolone sulfate, 16α-hydroxypregnenolone, and 20α-dihydropregnenolone did not differ between the GDM+ and GDM− groups (Table 1 and Table S1).

From the Δ^5^ androstane steroids (11 analytes), the levels of DHEA sulfate were higher in GDM+ women, the levels of five steroids (7α- and 7β-hydroxy-DHEA, 5-androstene-3β,7α,17β-triol, 5-androstene-3β,16α,17β-triol and its sulfate) were lower in the GDM+ group, and the levels of five steroids (DHEA, 7-oxo-DHEA, androstenediol, androstenediol sulfate, and 5-androstene-3β,7β,17β-triol) did not significantly differ between the groups (Table 1 and Table S1).

#### 3.2.2. Δ^4^ Steroids

From the Δ^4^ pregnanes (seven analytes), the levels of two steroids (conjugated 17α,20α-dihydroxy-4-pregnen-3-one and 20α-dihydroprogesterone) were lower in the GDM+ group, while the differences in levels of five steroids (progesterone, 17-hydroxyprogesterone, 17α,20α-dihydroxy-4-pregnene-3-one, conjugated 20α-dihydroprogesterone) did not reach statistical significance (Table 1 and Table S1).

However, the significant GDM × Stage interactions for progesterone, 16α-hydroxyprogesterone and 20α-dihydroprogesterone showed different relationships with gestational age (GA) for the GDM+ and GDM− groups in these steroids. While from week 24 to week 36 of gestation, the progesterone and 16α-hydroxyprogesterone levels were higher in the GDM+ group, the situation was different at labor. In progesterone levels, there was a fall between week 30–36 of gestation and labor for GDM+ group, while no such difference was found in GDM− group. The 16α-hydroxyprogesterone levels did not differ between week 30–36 of gestation and labor in the GDM+ group but continued to grow in the GDM− group (Table 1).

The levels of 20α-dihydroprogesterone gradually raised from week 24–28 to labor, but this raise was slower in the GDM+ group, so the levels were markedly lower at labor when compared with the GDM− group (Table 1).

From the Δ^4^ androstanes (5 analytes), the levels of two steroids (testosterone and 16α-hydroxytestosterone) were lower in the GDM+ group, while the levels of three steroids (androstenedione, conjugated testosterone, and conjugated epitestosterone) did not differ between the GDM+ and GDM− groups (Table 1 and Table S1).

#### 3.2.3. Balance between 17-Hydroxy-Steroids and Corresponding 17-Deoxy-Steroids

To estimate the influence of GDM and GA on activities of steroidogenic enzymes the appropriate product to precursor ratios (PPRs) were used.

Concerning the PPRs, the authors are aware that what happens in the blood (i.e., plasma levels) may not directly correlate with the function of the enzymes possibly related. Of course, the enzymes are present in different tissues that contribute in a different way to the plasma levels. However, numerous authors used the product-to-precursor ratios as surrogate markers for various diseases as well as for explanation of a variety of physiological and pathophysiological processes [49,50].

The relationships between PPRs that may reflect the activities of CYP17A1 in the 17-hydroxylase step and factors Group and Stage are shown in Table 2. From the 7 PPRs, those for the Δ^5^ pathway (17-hydroxy-pregnenolone/pregnenolone and 17-hydroxy-pregnenolone/pregnenolone, sulfates) did not differ between the GDM+ and GDM− groups. The corresponding PPRs for the Δ^4^ pathway (17-hydroxy-progesterone/progesterone) showed lower values for the GDM+ group, as did the PPR for 5α/β-reduced pregnanes, where the differences between the GDM+ and GDM− groups were even more pronounced (Table 2).

#### 3.2.4. Balance between Androstanes and Corresponding 17-Hydroxy-Steroids

The relationships between PPRs that may reflect the activities of CYP17A1 in the C17,20-lyase step and GDM are shown in Table 3. DHEA/17-hydroxypregnenolone significantly differs at labor only, when the values for the GDM+ group were markedly lower in comparison with the GDM− group. The PPR for the sulfated steroids did not differ between the groups, and the same was valid for the androstenedione/17-hydroxyprogesterone ratio in the Δ^4^ pathway. On the other hand, the PPRs for the 5α/β-reduced steroids were consistently higher in the GDM+ group (Table 3). The values of the DHEA/17-hydroxypregnenolone ratio decreased more rapidly in the GDM+ group (Table 3).

#### 3.2.5. Estrogens

Concerning the estrogens (6 analytes), four steroids (estrone sulfate, estradiol, estradiol sulfate, and estriol sulfate) were lower in the GDM+ group. The between-group differences in estrone and estriol did not reach significance (Table 1 and Table S1).

#### 3.2.6. 5α-Reduced Steroids and Steroid 5α-Reductases (SRD5As)

From the 5α-reduced-steroids (31 analytes), the levels of 18 steroids (allopregnanolone sulfate, isopregnanolone sulfate, conjugated 5α-pregnane-3α,20α-diol, conjugated 5α-pregnane-3β,20α-diol, 17-hydroxyallopregnanolone, 17-hydroxyallopregnanolone sulfate, 5α-pregnane-3α,17α,20α-triol, 5α-pregnane-3β,17α,20α-triol, and conjugated 5α-pregnane-3β,17α,20α-triol, androsterone, epiandrosterone sulfate, 5α-androstane-3α,17β-diol, conjugated 5α-androstane-3α,17β-diol, 5α-androstane-3β,17β-diol, conjugated 5α-androstane-3β,17β-diol, 11β-hydroxyandrosterone, 11β-hydroxyandrosterone sulfate, and 11β-hydroxyepiandrosterone) were lower in the GDM+ group. 3α,5α-Tetrahydrocorticosterone levels were higher in the GDM+ group. The levels of 12 steroids (5α-dihydroprogesterone, allopregnanolone, isopregnanolone, 5α,20α-tetrahydroprogesterone, conjugated 5α,20α-tetrahydroprogesterone, 5α-pregnane-3α,20α-diol, 5α-pregnane-3β,20α-diol, and conjugated 5α-pregnane-3α,17α,20α-triol, 5α-androstane-3,17-dione, androsterone sulfate, epiandrosterone, and 11β-hydroxyepiandrosterone sulfate) did not significantly differ between the groups (Table 1 and Table S1).

The seven PPRs that may reflect the 5α-reductase (SRD5As) activities are presented in Table 4.

#### 3.2.7. 5β-Reduced Steroids and Steroid 5β-Reductase (AKR1D1)

The relationships between 5β-reduced steroids and GDM were inconsistent (Table 1 and Table S1) and none of the PPRs that may reflect AKR1D1 were significantly associated with GDM (Table S3).

#### 3.2.8. The Balance between 17β-hydroxy- and 17-oxo-steroids

From the nine ratios that may reflect the balance between 17β-hydroxy- and 17-oxo-steroids (Table 5), three ratios showed lower values in the GDM+ group than in the GDM− group (androstenediol/DHEA, testosterone/androstenedione, estradiol/estrone), four ratios did not differ between the groups (5α-dihydrotestosterone/5α-androstane-3,17-dione, 5α-androstane-3α,17β-diol/androsterone, 5α-androstane-3β,17β-diol/epiandrosterone, 11β-hydroxy-testosterone/11β-hydroxyandrostenedione), and two ratios showed higher values in the GDM+ group (5-androstene-3β,7α,17β-triol/7α-hydroxy-DHEA, 5-androstene-3β,7β,17β-triol/7β-hydroxy-DHEA) (Table 5).

#### 3.2.9. 16α-Hydroxylation

Concerning the steroid 16α-hydroxylation, the differences between the GDM+ and GDM− groups were not consistent (Table S4).

#### 3.2.10. The Balance between Conjugated and Unconjugated Steroids

To assess the effect of GDM and GA on the balance between conjugated and unconjugated steroids the ratios of steroid conjugates to unconjugated steroids (C/F) were evaluated. No consistent trend was observed concerning the relations between C/Fs and GDM status. However, despite the relatively low overall consistency, the results were more consistent for GABAergic steroids showing lower C/F in GDM+ women (allopregnanolone, pregnanolone, androsterone, 5α-androstane-3α,17β-diol) (Table 6).

#### 3.2.11. 3β-Hydroxysteroid Dehydrogenases (HSD3Bs)

From the seven PPRs that may reflect the activities of HSD3Bs, two of them (progesterone/pregnenolone, 16α-hydroxytestosterone/5-androstene-3β,16α,17β-triol) showed lower values in the GDM+ group, 4 PPRs did not significantly differ between the groups (20α-dihydroprogesterone/20α-dihydropregnenolone, 17-hydroxyprogesterone/17-hydroxypregnenolone, androstenedione/DHEA, testosterone/androstenediol) and 16α-hydroxyprogesterone/16α-hydroxy-pregnenolone values were higher in the GDM+ group (Table 7).

#### 3.2.12. Conversion of Adrenal Androgens to Their 7α/β-Hydroxy-Derivatives

From the four PPRs that may reflect the conversion of adrenal androgens to their 7α/β-hydroxy-derivatives, the ratios of 7α-hydroxy-DHEA/DHEA and 7β-hydroxy-DHEA/DHEA showed lower values in the GDM+ group when compared with the GDM− group. No significant between-group differences were found in 5-androstene-3β,7α,17β-triol/androstenediol and androstene-3β,7α,17β-triol/androstenediol ratios (Table 8).

#### 3.2.13. Type 1 11β-Hydroxysteroid Dehydrogenase (HSD11B1)

From the five PPRs that may reflect the HSD11B1 activity, the 7β-hydroxy-DHEA/7-oxo-DHEA ratio was significantly lower in the GDM+ group, 3 PPRs (7-oxo-DHEA/7α-hydroxy-DHEA, 5-androstene-3β,7β,17β-triol/5-androstene-3β,7α,17β-triol, 7β-hydroxy-DHEA/7α-hydroxy-DHEA) did not differ between the groups. The cortisol/cortisone ratio showed higher values in the GDM+ group when compared with the GDM− group (Table 9).

## 4. Discussion

### 4.1. Comparison of Alterations in Maternal and Fetal Steroidome Related to GDM

The data indicates that the effect of GDM on the fetal steroidome (Figure 1, Table S2) is considerably weaker compared to maternal steroidogenesis (Table 1 and Table S1). Since in the cord blood there were three 5α/β-reduced metabolites of 17-hydroxyprogesterone and one 5α-reduced androgen, which showed lower levels in the GDM+ group, it can be concluded that GDM may be associated with lower activity of CYP17A1 in the C17-hydroxylase step at least (Figure 1, Table S2). Whereas the placenta has low activity of CYP17A1, it cannot substantially affect the percentage of 5α/β-reduced 17-deoxy-, 17-hydroxy-pregnanes and 5α/β-reduced androstanes during transplacental passage from mother into the fetus. However, it readily metabolizes Δ^5^ and Δ^4^ steroids. Since none of the Δ^5^ or Δ^4^ steroids in cord blood showed significant differences between the GDM+ and GDM− groups (Table S2), the differences in the 5α/β-reduced metabolites of 17-hydroxyprogesterone and one 5α-reduced androgen may be considered as a sign of reduced activity of maternal and/or placental CYP17A1 in GDM+ group (see [51]).

### 4.2. Alterations in Maternal Steroidome Related to GDM

#### 4.2.1. Higher Levels of Pregnenolone in Late Gestation in the GDM+ Group

Interestingly, in the group of steroids that were consistently higher in the circulation of GDM+ women in all stages of the study, there was pregnenolone whose levels did not differ in cord blood between the GDM+ and GDM− groups (Table 1 and Table S2). Unlike pregnenolone, the levels of PregS, 17-hydroxypregnenolone and its sulfate did not differ between the GDM+ and GDM− groups (Table 1 and Table S1). This could be explained by the increased CYP11A1 activity at reduced activity of both CYP17A1 and SULT2A1 in mothers with GDM. However, the 17-hydroxypregnenolone to pregnenolone ratio did not differ between the GDM+ and GDM− groups (Table 2). The lower ratio of sulfated to unsulfated pregnenolone between week 24–28 and week 30–36 of gestation (Table 6) was consistent with reduced SULT2A1 activity in GDM+ women in adrenal *zona fasciculata* (ZF) where the PregS is primarily produced.

It is known that both PregS and DHEAS act on various ionotropic receptors, which differently influence the glucose homeostasis. Because PregS levels did not differ between the GDM+ and GDM− groups, while DHEAS levels were slightly but significantly higher in women with GDM+, a slightly diabetogenic effect of sulfated Δ^5^ steroids can be assumed in late gestation.

#### 4.2.2. The Increase of Diabetogenic Effects with Advancing GA Is Associated with Mounting Progesterone Levels

Progesterone in pregnancy is of placental origin and is synthesized partly from the maternal LDL and partly from the fetal PregS, and then secreted to both maternal and fetal circulation [52,53]. Concerning the extremely elevated levels of progesterone in pregnant women and so much more in the fetus (Table 1), it is evident that even maternal progesterone levels are more dependent on fetal and placental steroidogenesis than on maternal production.

As already mentioned, progesterone is a central regulator of β-cell proliferation in response to metabolic challenges. Therefore progesterone increase between week 24–28 and week 30–36 of gestation may play a role in the suppression of IS and down-regulation of β-cell function in the later stages of gestation [12,27]). In addition, progesterone suppresses the IS in the β-cells via inhibition of TRPM3 channels [15]. Even if the effects of progesterone on IS may not be unidirectional [16,17], our data support the concept that the intensification of diabetogenic effects with advancing GA is associated with mounting progesterone levels and the predominant progesterone effect appears to be diabetogenic, as the progesterone levels are consistently higher in the GDM+ group in week 24–28 and week 30–36 of gestation when compared with the GDM− group. However, the situation is reversed at labor. A trend towards lower levels of 16α-hydroxyprogesterone and 20α-dihydroprogesterone was also observed in the GDM+ group at labor (Table 1). In addition to its diabetogenic effects, progesterone as cortisol triggers immunoregulatory signals in T cells (see [51]).

#### 4.2.3. Lower Testosterone Levels in the GDM+ Group Are Associated with Lower AKR1C3 Activity

Similar dependence on GA, like the Δ^5^ steroids, was exhibited also in the Δ^4^ androstanes (Table 1 and Table S1). These steroids are primarily synthesized in the sequence DHEA, androstenedione (catalyzed by HSD3B2), testosterone (catalyzed by HSD17B3 and AKR1C3) or in the sequence DHEA, androstenediol (catalyzed by AKR1C3, and HSD17B3), testosterone (catalyzed by HSD3B2) [49,50]. The levels of testosterone were consistently lower in the GDM+ group in contrast to the levels of androstenedione and androstenediol (Table 1 and Table S1). The androstenedione/DHEA ratio and testosterone/androstenediol ratio did not differ between the groups (Table 5) but the testosterone/androstenedione and androstenediol/DHEA ratios were lower in the GDM+ group (Table 5). Since the HSD17B3 is a testicular enzyme, while the AKR1C3 is widely expressed in various tissues and explicitly in adipocytes (and further tissues such as the digestive tract, smooth muscle, pancreas and to a much lesser extent also in adrenal *zona reticularis* (ZR)), the aforementioned data point to the reduced AKR1C3 activity in the GDM+ group. Since the aforementioned tissues play an important role in the pathophysiology of DM2, these findings may be relevant for GDM pathophysiology.

#### 4.2.4. Lower Levels of Estrogens and GDM Pathophysiology

From the total number of six estrogens, the levels of four of them, including the bioactive estradiol, were lower in the GDM+ group (Table 1 and Table S1).

Concerning the PPRs that may reflect the activity of aromatase (CYP19A1), neither the estrone/androstenedione ratio nor the estradiol/testosterone ratio [49,50] differed significantly between the GDM+ and GDM− groups (Table S5). Instead, the estradiol/estrone ratio that may reflect the balance between placental enzymes such as reductive HSD17B1 and oxidative HSD17B2 [49,50] showed lower values in the GDM+ group (Table 5). Hence, this shift may be responsible for the relative estradiol deficit in the GDM+ group. Estradiol is an important antidiabetic steroid operating via binding to nuclear receptors as well as via modulation of ion channels controlling the secretion of pancreatic hormones [12,27,28,29,30,31,32]. Therefore, the estradiol deficiency in the GDM+ group may be an important component participating in the pathophysiology of GDM.

#### 4.2.5. Lower Activity of CYP17A1 in the Hydroxylase Step at Higher Activity in the Lyase Step in GDM+ Women

While the PPRs that may reflect the CYP17A1 activity in the hydroxylase step for Δ^5^ pregnanes did not significantly differ between the GDM+ and GDM− groups, they were significantly lower for Δ^4^ pregnanes and 5α/β-reduced pregnanes (Table 2). Alternatively, the PPRs that may reflect the CYP17A1 activity for the 5α/β-reduced steroids in the lyase step were markedly higher, particularly in week 24–28 of gestation (Table 3). These data outline the importance of the “backdoor pathway” in the metabolism of pregnane steroids, as the 5α-reduced- (and possibly 5β-reduced), 3α-hydroxy-pregnanes are excellent substrates with high affinity for CYP17A1 (in both hydroxylase and lyase steps) [54]. Moreover, these more stable reduced catabolites appear to be better indicators of the CYP17A1 activities than the Δ^4^ and Δ^5^ steroids. The functional activity of the “backdoor pathway” in placenta was recently hypothesized by Karahoda et al. [51].

#### 4.2.6. Lower Conjugation of Some Neuroactive Pregnanolone Isomers and Pregnenolone in GDM+ Women

Conjugation of pregnane steroids may occur mainly via sulfation or glucuronidation pathways, and the proportions between the resulting conjugates may differ from steroid to steroid from almost 100% to about 50% of sulfates and/or disulfates [40,41,45,46].

In terms of the biological effects of neuroactive steroids, the activity of the steroidogenic enzymes responsible for steroid conjugation has a major influence. For instance, the sulfation of allopregnanolone blocks its neuroinhibitory effect on GABA_A_R [55], glycine receptors (GlyR) [56], and α-amino-3-hydroxy-5-methyl-4-isoxazolepropionic acid receptors (AMPAR) [19], and forms a slightly excitatory substance via positive modulation of N-methyl-D-aspartate receptors (NMDAR) [57]. On the other hand, the sulfation of allopregnanolone stops its excitatory effect in fetal rat hypothalamic neurons via L-type voltage-gated calcium channels (VGCC) [58]. Besides, the sulfation of pregnanolone reverses its neuroinhibitory activity on GABA_A_R [23,59] but forms another neuroinhibitory agent, pregnanolone sulfate, negatively modulating NMDAR [60,61] and AMPAR [18]. In addition, the pregnanolone sulfate also inhibits the permeability of short transient receptor potential channel 5 (TRPC5) [62], influencing the fear-related behavior [63]. The sulfation of the steroid isopregnanolone amplifies its slight neuroexcitatory effect (via negative modulation of GABA_A_R) [64,65].

Despite the low uniformity in the balance between free and conjugated steroids, the results are more consistent in some groups of steroids, such as pregnanolone isomers (Table 6). Concerning the lower conjugation of these steroids in GDM+ women (pregnenolone, allopregnanolone, isopregnanolone, pregnanolone, androsterone, epiandrosterone, and 5α-androstane-3α,17β-diol), the data indicate a prevalent trend to lower clearance for neuroinhibitory (neuroprotective) positive modulators of GABA_A_R and GlyR (allopregnanolone, pregnanolone, androsterone, 5α-androstane-3α,17β-diol) on one hand, but reduced formation of efficient excitatory sulfated steroids (pregnenolone sulfate, isopregnanolone sulfate) in GDM+ compared to the GDM− group [23,55,56,59,64,65,66,67,68] on the other.

#### 4.2.7. Lowered 5α-Reductase Activities in GDM+ Women

SRD5As activities represent the rate-limiting step in the synthesis of neurosteroids and generally in the steroid catabolism [69,70]. The most PPRs that may reflect the SRD5As activities indicate lower values in GDM+ women than in controls (Table 4). The isoforms of SRD5As are expressed in numerous tissues including placenta [71,72]. Relative to tissue weight, the human placenta expresses SRD5A1 around 1000 times more than the testis (see [51]). The highest extraplacental expression of the SRD5A1 isoform has been reported in the fetal brain [72], which is likely related to increased fetal brain demands for synthesis of the neuroprotective GABAergic steroid allopregnanolone [70]. The SRD5A2 isoform is most expressed in the maternal liver [72].

Concerning the relationships between gestational diabetes and SRD5As, the data in the literature is scarce. Manjunath-Gowda reported results indicating reduced SRD5As activity in GDM+ women compared to controls [73]. Regarding the associations between SRD5As and DM2, Traish et al. found that the suppression of SRD5As activities by finasteride and dutasteride weaken the clearance of glucocorticoids (potentiating IR, diabetes and vascular disease) [69]. Other authors published that a dual inhibition of SRD5As, but not inhibition of SRD5A2 alone, modulates insulin sensitivity in human peripheral tissues rather than liver [74]. Nasiri et al. reported that SRD5A2 overexpression reduced the effects of cortisol to suppress lipogenesis, but SRD5A2 inhibition increased cortisol action [75]. Our data indicating reduced SRD5As activities in the GDM+ group are consistent with the above reports, and imply that reduced SRD5As activities may contribute to the pathophysiology of both DM2 and GDM.

#### 4.2.8. Reduced 7-Hydroxylation of DHEA and Attenuated Competition of 7-Oxygenated Androstanes with Cortisone for HSD11B1 May Participate in the Pathophysiology of GDM

Endogenous 7-hydroxy-Δ^5^-steroids and their synthetic analogues are potential agents to treat autoimmune diseases [37]. From the immunoprotective and anti-inflammatory Δ^5^ 7α/β-androstane steroids [76], the levels of 7α-hydroxy-DHEA, 7β-hydroxy-DHEA, and 5-androstene-3β,7α,17β-triol were significantly lower in the GDM+ group (Table 1). As regards the PPRs that may reflect the activities of 7α/β-hydroxylating enzymes [36,77], those for DHEA were significantly lower in the GDM+ group (Table 8). It should be pointed out that the interconversion in the sequence 7α-hydroxy-DHEA, 7-oxo-DHEA, 7β-hydroxy-DHEA is catalyzed by the same enzyme (HSD11B1), which converts the inactive cortisone to the bioactive glucocorticoid cortisol [36]. From the PPRs that may reflect the HSD11B1 activity, the cortisol/cortisone ratio was higher in the GDM+ group when compared with the GDM− group, but another PPRs that may reflect the HSD11B1 activity 7β-Hydroxy-DHEA/7-oxo-DHEA was lower in the GDM+ group (Table 9). While cortisol levels did not differ between the groups, cortisone levels were lower in the GDM+ group (Table 1 and Table S1). Taken together, this data could be explained as follows. The lower activity of the CYP7B1 enzyme results in reduced production of 7-oxygenated steroids, which may lead to decreased competition of these substances for active sites of the enzyme HSD11B1 with cortisone. As a result, the balance between cortisone and cortisol is shifted towards the active glucocorticoid cortisol. Since the bioactive cortisol, in contrast to inactive cortisone, belongs to diabetogenic steroids, the suggested mechanism may participate in GDM pathophysiology [78].

## 5. Conclusions

The present data show that GDM−related steroidomic changes are evident in the maternal blood, but not in the mixed umbilical blood. This suggests a decisive role for the maternal compartment in GDM pathophysiology, although concentrations of neuroactive steroids that can affect glucose homeostasis are generally significantly higher in mixed cord blood compared to maternal blood.

Concerning the levels of sulfated Δ^5^ steroids influencing the pathophysiology of GDM, our data indicates either a balanced or slightly pro-diabetogenic effect of these neuroactive substances.

In line with reports from other authors, our results demonstrate that the increase of diabetogenic effects is associated with mounting progesterone levels with the approaching term.

The GDM+ group exhibits lower testosterone levels, most probably due to attenuated AKR1C3 activity. Lower levels of antidiabetic estradiol in the GDM+ group may be associated with a shift from the reductive HSD17B1 activity to the activity of oxidative HSD17B2 isoform. The data also indicate that the suppressed production of antidiabetic estradiol may be an important component in the pathophysiology of GDM.

The activity of CYP17A1 is an essential step of cortisol biosynthesis. Therefore, the observed trend to lower activity of CYP17A1 in the hydroxylase step in the GDM+ group may indicate some disruption in cortisol production.

The mostly decreasing activity in the CYP17A1 lyase step in GDM+ group women may be associated with increasing levels of estradiol towards term as estradiol suppresses the synthesis of adrenal androgens in the maternal compartment. The data regarding the CYP17A1 activity outline the importance of the “backdoor pathway” in the metabolism of C21 steroids, as the reduced pregnanes are excellent substrates with a high affinity to this enzyme [54]. These metabolically stable analytes appear to be better indicators of the CYP17A1 activity in both hydroxylase- and lyase steps than the Δ^5^ and Δ^4^ steroids.

The lower conjugation of some physiologically active steroids in GDM+ women indicates a prevalent trend to lower clearance for neuroinhibitory (and mostly neuroprotective) positive modulators of GABA_A_R and lower formation of excitatory (and sometimes excitotoxic) sulfated steroids as pregnenolone sulfate and isopregnanolone sulfate in the GDM+ group. Moreover, the rising conjugation of neuroinhibitory GABAergic pregnanes and pregnancy-stabilizing steroids 5β-reduced pregnanes towards labor may also be connected with functional progesterone withdrawal.

Women with GDM showed reduced SRD5As activities, which may play a role in the pathophysiology of GDM in addition to DM2. However, in contrast to the diabetogenic effect of SRD5As, their overexpression might contribute to higher production of GABAergic (and neuroprotective) 5α-reduced steroids.

The lower activity of the CYP7B1 enzyme results in reduced production of 7-oxygenated adrenal androgens, which may lead to decreased competition of these substances for active sites of the enzyme HSD11B1 with cortisone. As a result, the balance between cortisone and cortisol is shifted towards the active glucocorticoid cortisol. Since the bioactive cortisol in contrast to inactive cortisone belongs to diabetogenic steroids, the suggested mechanism may participate in GDM pathophysiology. The absent difference between the GDM+ and GDM− groups in cortisol levels may be explained with the suppressed CYP17A1-lyase activity in the GDM+ group and reduced of CYP7B1 activity in this group. Regarding the dependence on GA, the production of the immunoprotective 7-deoxy- and the more active 7-oxygenated adrenal androgens mounts towards labor, which may stimulate immunoprotective effects with the approaching term.

In conclusion, this study produces evidence that the maternal but not the fetal steroidome is substantially altered in women with GDM, which may have a number of consequences concerning the GDM pathophysiology.

## Figures and Tables

**Table 1:** Profiles of steroids from week 24 of gestation to labor, which were significantly related to GDM.

Steroid	GDM	GDM × Stage		
		Week 24−28 of Gestation (a)	Week 30−36 of Gestation (b)	Labor (c)
Pregnenolone [nM]	−	4.7 (4.3, 5.2)	5.2 (4.8, 5.7)	3.8 (3.4, 4.2)
	+	6 (5.5, 6.5) *	6.3 (5.8, 6.8) *	5.2 (4.7, 5.6) *
		G (WP): F = 21.8, *p* < 0.001; S: F = 8.9, *p* < 0.001; G × S: F = 0.4, *p* = 0.7; GDM− c < a, c < b; GDM+ c < b		
16α-Hydroxypregnenolone sulfate [nM]	−	39 (30, 52)	68 (52, 91)	110 (83, 150)
	+	26 (20, 34)	50 (38, 65)	89 (67, 120)
		G (UP): F = 4.2, *p* = 0.044; S: F = 18.2, *p* < 0.001; G × S: F = 0.2, *p* = 0.851; GDM− c > a; GDM+ b > a, c > a, c > b		
20α-Dihydropregnenolone sulfate [nM]	−	280 (270, 300)	280 (260, 290)	410 (380, 440)
	+	320 (300, 340)	320 (300, 340) *	470 (440, 500)
		G(UP): F = 11.8, *p* < 0.001; S: F = 51.5, *p* < 0.001; G × S: F = 0.1, *p* = 0.892; GDM− c > a, c > b; GDM+ c > a, c > b		
DHEA sulfate [μM]	−	1.3 (1.2, 1.4)	1.2 (1.1, 1.3)	1.6 (1.5, 1.8)
	+	1.5 (1.4, 1.6)	1.3 (1.2, 1.4)	1.8 (1.7, 1.9)
		G(UP): F = 4.7, *p* = 0.032; S: F = 21.8, *p* < 0.001; G × S: F = 0.1, *p* = 0.865; GDM− c > a, c > b; GDM+ c > a, c > b		
7α-Hydroxy-DHEA [nM]	−	0.31 (0.26, 0.37)	0.38 (0.32, 0.45)	0.87 (0.75, 1)
	+	0.22 (0.18, 0.26)	0.29 (0.24, 0.34)	0.72 (0.61, 0.84)
		G(UP): F = 7.7, *p* = 0.006; S: F = 53.8, *p* < 0.001; G × S: F = 0.2, *p* = 0.862; GDM− c > a, c > b; GDM+ c > a, c > b		
7β-Hydroxy-DHEA [nM]	−	0.34 (0.3, 0.38)	0.36 (0.31, 0.4)	0.52 (0.47, 0.58)
	+	0.24 (0.2, 0.27) *	0.29 (0.25, 0.33)	0.41 (0.36, 0.46) *
		G(WP): F = 13.4, *p* < 0.001; S: F = 17.6, *p* < 0.001; G × S: F = 0.3, *p* = 0.768; GDM− c > a, c > b; GDM+ c > a, c > b		
5-Androstene-3β,7α,17β-triol [nM]	−	0.13 (0.11, 0.15)	0.14 (0.12, 0.16)	0.34 (0.3, 0.39)
	+	0.11 (0.095, 0.12)	0.13 (0.12, 0.15)	0.23 (0.2, 0.26) *
		G(UP): F = 8, *p* = 0.006; S: F = 57.9, *p* < 0.001; G × S: F = 2.6, *p* = 0.082; GDM− c > a, c > b; GDM+ c > a, c > b		
5-Androstene-3β,16α,17β-triol [nM]	−	0.63 (0.56, 0.7)	0.9 (0.81, 1)	1.7 (1.5, 1.8)
	+	0.52 (0.46, 0.58)	0.89 (0.8, 1)	1.2 (1.1, 1.3) *
		G(UP): F = 8.8, *p* = 0.004; S: F = 74.8, *p* < 0.001; G × S: F = 2.9, *p* = 0.059; GDM− b > a, c > a, c > b; GDM+ b > a, c > a, c > b		
5-Androstene-3β,16α,17β-triol sulfate [nM]	−	93 (83, 100)	140 (130, 160)	260 (240, 290)
	+	78 (70, 86)	120 (110, 130)	230 (200, 250)
		G(UP): F = 7.7, *p* = 0.007; S: F = 116.8, *p* < 0.001; G × S: F = 0, *p* = 0.989; GDM− b > a, c > a, c > b; GDM+ b > a, c > a, c > b		
Progesterone [nM]	−	280 (250, 310)	400 (360, 440)	360 (320, 400)
	+	360 (320, 390) *	500 (450, 540) *	290 (260, 320) *
		G: F = 2.4, *p* = 0.125; S: F = 18.7, *p* < 0.001; G × S (WP): F = 7.3, *p* = 0.001; GDM− b > a, c > a; GDM+ b > a, c < a, c < b		
Conjugated 17α,20α-dihydroxy-4-pregnen-3-one [nM]	−	3.6 (3.1, 4.3)	4.6 (3.9, 5.5)	9.4 (7.9, 11)
	+	2.7 (2.3, 3.2)	3.2 (2.7, 3.7) *	6.2 (5.2, 7.3) *
		G(WP): F = 15.4, *p* < 0.001; S: F = 34.3, *p* < 0.001; G × S: F = 0.1, *p* = 0.876; GDM− c > a, c > b; GDM+ c > a, c > b		
20α-Dihydroprogesterone [nM]	−	49 (46, 53)	85 (79, 92)	120 (110, 130)
	+	51 (48, 54)	78 (73, 83)	91 (86, 98) *
		G(UP): F = 8, *p* = 0.006; S: F = 142, *p* < 0.001; G × S(WP): F = 6.3, *p* = 0.003; GDM− b > a, c > a, c > b; GDM+ b > a, c > a, c > b		
Testosterone [nM]	−	2.3 (2.1, 2.5)	2.5 (2.3, 2.7)	3.6 (3.3, 4)
	+	2 (1.8, 2.2)	2 (1.8, 2.1) *	3.1 (2.8, 3.4)
		G(WP): F = 12.8, *p* < 0.001; S: F = 32.8, *p* < 0.001; G × S: F = 0.3, *p* = 0.723; GDM− c > a, c > b; GDM+ c > a, c > b		
16α-Hydroxytestosterone [nM]	−	4.9 (3.8, 6.1)	8.2 (6.7, 9.9)	9.3 (7.7, 11)
	+	3.5 (2.6, 4.5)	6.6 (5.4, 8.1)	6.5 (5.2, 7.9)
		G(UP): F = 6.5, *p* = 0.012; S: F = 11.3, *p* < 0.001; G × S: F = 0.2, *p* = 0.848; GDM− b > a, c > a; GDM+ b > a, c > a		
Estrone sulfate [nM]	−	96 (77, 120)	110 (87, 140)	190 (150, 240)
	+	70 (56, 87)	98 (79, 120)	120 (97, 150) *
		G(UP): F = 5.6, *p* = 0.02; S: F = 8.9, *p* < 0.001; G × S: F = 0.6, *p* = 0.528; GDM− c > a, c > b; GDM+ c > a		
Estradiol [nM]	−	66 (54, 79)	90 (74, 110)	66 (54, 80)
	+	47 (38, 57)	71 (58, 85)	48 (40, 59)
		G(UP): F = 7, *p* = 0.009; S: F = 4.8, *p* = 0.01; G × S: F = 0.1, *p* = 0.945; GDM+ b > a		
Estradiol sulfate [nM]	−	34 (29, 39)	49 (42, 56)	55 (48, 63)
	+	25 (22, 29) *	36 (31, 41) *	30 (26, 34) *
		G(WP): F = 26.4, *p* < 0.001; S: F = 8.5, *p* < 0.001; G × S: F = 1.8, *p* = 0.168; GDM− b > a, c > a; GDM+ b > a		
Estriol sulfate [nM]	−	170 (150, 200)	280 (240, 320)	360 (310, 410)
	+	120 (100, 130) *	170 (150, 200) *	280 (250, 330)
		G(WP): F = 20.4, *p* < 0.001; S: F = 37.2, *p* < 0.001; G × S: F = 0.9, *p* = 0.405; GDM− b > a, c > a; GDM+ b > a, c > a, c > b		
Allopregnanolone sulfate [μM]	−	0.84 (0.76, 0.92)	1.1 (1, 1.2)	1.2 (1.1, 1.3)
	+	0.58 (0.53, 0.63) *	0.98 (0.9, 1.1)	1.1 (1, 1.2)
		G(WP): F = 14.5, *p* < 0.001; S: F = 37.4, *p* < 0.001; G × S: F = 3.3(WP), *p* = 0.041; GDM− b > a, c > a; GDM+ b > a, c > a		
Isopregnanolone sulfate [nM]	−	470 (420, 520)	610 (550, 690)	750 (670, 840)
	+	330 (300, 370) *	550 (490, 610)	630 (560, 700)
		G(UP): F = 11.3, *p* = 0.001; S: F = 29, *p* < 0.001; G × S: F = 1.2, *p* = 0.301; GDM− b > a, c > a; GDM+ b > a, c > a		
Conjugated pregnanolone [nM]	−	480 (440, 530)	640 (580, 700)	690 (630, 770)
	+	380 (350, 420) *	640 (580, 700)	780 (710, 870)
		G: F = 0.6, *p* = 0.439; S: F = 40.2, *p* < 0.001; G × S(WP): F = 4, *p* = 0.021; GDM− b > a, c > a; GDM+ b > a, c > a, c > b		
Conjugated epipregnanolone [nM]	−	130 (120, 140)	160 (150, 180)	200 (180, 220)
	+	97 (88, 110) *	140 (130, 160)	160 (140, 170) *
		G(WP): F = 14.3, *p* < 0.001; S: F = 22.9, *p* < 0.001; G × S: F = 0.6, *p* = 0.559; GDM− b > a, c > a; GDM+ b > a, c > a		
Conjugated 5α-pregnane-3α,20α-diol [μM]	−	8.1 (7.5, 8.7)	11 (9.8, 11)	14 (13, 15)
	+	6.1 (5.7, 6.5) *	9.3 (8.7, 10)	12 (11, 13)
		G(WP): F = 20, *p* < 0.001; S: F = 76, *p* < 0.001; G × S: F = 1.7, *p* = 0.194; GDM− b > a, c > a, c > b; GDM+ b > a, c > a, c > b		
Conjugated 5α-pregnane-3β,20α-diol [μM]	−	19 (17, 20)	26 (24, 29)	31 (29, 34)
	+	15 (14, 17) *	23 (21, 25)	28 (26, 30)
		G(UP): F = 10.8, *p* = 0.001; S: F = 51.2, *p* < 0.001; G × S: F = 0.3, *p* = 0.734; GDM− b > a, c > a, c > b; GDM+ b > a, c > a, c > b		
5β-Pregnane-3α,20α-diol [nM]	−	5.8 (5.4, 6.3)	7.4 (6.8, 8.1)	7.3 (6.7, 8)
	+	7.2 (6.7, 7.9) *	8.8 (8, 9.6)	7.1 (6.5, 7.7)
		G(UP): F = 6.2, *p* = 0.014; S: F = 7.2, *p* = 0.001; G × S: F = 2.7, *p* = 0.073; GDM− b > a, c > a; GDM+ b > a, c < b		
17-Hydroxyallopregnanolone [nM]	−	0.34 (0.29, 0.4)	0.54 (0.46, 0.63)	0.59 (0.51, 0.68)
	+	0.17 (0.15, 0.2) *	0.39 (0.33, 0.45) *	0.38 (0.33, 0.44) *
		G(WP): F = 30.5, *p* < 0.001; S: F = 25.1, *p* < 0.001; G × S: F = 1, *p* = 0.357; GDM− b > a, c > a; GDM+ b > a, c > a		
17-Hydroxyallopregnanolone sulfate [nM]	−	11 (10, 12)	17 (15, 18)	22 (20, 24)
	+	8 (7.5, 8.7) *	13 (12, 14) *	18 (17, 20)
		G(WP): F = 28.7, *p* < 0.001; S: F = 78.9, *p* < 0.001; G × S: F = 1.5, *p* = 0.219; GDM− b > a, c > a, c > b; GDM+ b > a, c > a, c > b		
17-Hydroxypregnanolone [nM]	−	0.81 (0.72, 0.89)	1 (0.92, 1.1)	1.2 (1.1, 1.4)
	+	0.68 (0.61, 0.75)	0.91 (0.82, 1)	0.91 (0.82, 1) *
		G(UP): F = 12.2, *p* < 0.001; S: F = 14.8, *p* < 0.001; G × S: F = 1.2, *p* = 0.294; GDM− b > a, c > a, c > b; GDM+ b > a, c > a		
Conjugated 17-hydroxy-pregnanolone [nM]	−	44 (40, 48)	68 (62, 74)	110 (96, 120)
	+	38 (36, 41)	58 (53, 63)	95 (86, 100)
		G(UP): F = 8, *p* = 0.006; S: F = 121.1, *p* < 0.001; G × S: F = 0.2, *p* = 0.854; GDM− b > a, c > a, c > b; GDM+ b > a, c > a, c > b		
5α-Pregnane-3α,17α,20α-triol [nM]	−	0.18 (0.15, 0.21)	0.19 (0.16, 0.22)	0.25 (0.21, 0.29)
	+	0.1 (0.089, 0.12) *	0.15 (0.13, 0.18)	0.18 (0.15, 0.21) *
		G(WP): F = 15.5, *p* < 0.001; S: F = 8.4, *p* < 0.001; G × S: F = 1.2, *p* = 0.296; GDM− c > a; GDM+ b > a, c > a		
5α-Pregnane-3β,17α,20α-triol [pM]	−	110 (90, 130)	130 (120, 160)	150 (130, 170)
	+	73 (61, 87) *	110 (92, 130)	110 (96, 130)
		G(UP): F = 10.1, *p* = 0.002; S: F = 6.5, *p* = 0.002; G × S: F = 0.2, *p* = 0.829; GDM− c > a; GDM+ b > a, c > a		
Conjugated 5α-pregnane-3β,17α,20α-triol [nM]	−	3.6 (2.8, 4.5)	5.2 (4.1, 6.7)	7.7 (6, 9.9)
	+	2.1 (1.7, 2.7) *	2.8 (2.2, 3.5) *	6.2 (4.8, 8.1)
		G(UP): F = 10.9, *p* = 0.001; S: F = 16.1, *p* < 0.001; G × S: F = 0.9, *p* = 0.393; GDM− c > a; GDM+ c > a, c > b		
5β-Pregnane-3α,17α,20α-triol [nM]	−	3.1 (2.7, 3.4)	3.4 (3, 3.8)	4.5 (4, 5)
	+	2.4 (2.1, 2.7) *	3.3 (2.9, 3.7)	3.9 (3.4, 4.3)
		G(UP): F = 4.7, *p* = 0.032; S: F = 15.5, *p* < 0.001; G × S: F = 1.1, *p* = 0.334; GDM− c > a, c > b; GDM+ b > a, c > a		
Conjugated 5β-pregnane-3α,17α,20α-triol [nM]	−	250 (220, 290)	380 (330, 440)	890 (740, 1100)
	+	230 (200, 270)	270 (240, 310) *	500 (430, 590) *
		G(WP): F = 13.5, *p* < 0.001; S: F = 47.2, *p* < 0.001; G × S: F = 2.1, *p* = 0.131; GDM− b > a, c > a, c > b; GDM+ c > a, c > b		
Androsterone [nM]	−	0.46 (0.43, 0.5)	0.52 (0.48, 0.56)	0.63 (0.58, 0.69)
	+	0.39 (0.36, 0.43) *	0.45 (0.41, 0.48)	0.53 (0.49, 0.58) *
		G(UP): F = 12.3, *p* < 0.001; S: F = 15.1, *p* < 0.001; G × S: F = 0, *p* = 0.979; GDM− c > a, c > b; GDM+ c > a, c > b		
Epiandrosterone sulfate [nM]	−	110 (110, 120)	100 (96, 110)	130 (130, 140)
	+	110 (100, 120)	93 (88, 98)	120 (110, 130)
		G(UP): F = 4.9, *p* = 0.029; S: F = 26.5, *p* < 0.001; G × S: F = 0.9, *p* = 0.409; GDM− c > a, c > b; GDM+ b < a, c > b		
Etiocholanolone sulfate [nM]	−	31 (29, 34)	30 (27, 33)	42 (39, 46)
	+	36 (33, 40)	32 (29, 35)	50 (46, 55)
		G(UP): F = 6.7, *p* = 0.011; S: F = 23.8, *p* < 0.001; G × S: F = 0.3, *p* = 0.722; GDM− c > a, c > b; GDM+ c > a, c > b		
5α-Androstane-3α,17β-diol [nM]	−	0.14 (0.13, 0.15)	0.16 (0.15, 0.18)	0.18 (0.16, 0.19)
	+	0.14 (0.13, 0.15)	0.15 (0.14, 0.16)	0.14 (0.13, 0.15) *
		G(WP): F = 9.4, *p* = 0.003; S: F = 2.3, *p* = 0.103; G × S: F = 2.5, *p* = 0.09; GDM− c > a		
Conjugated 5α-androstane-3α,17β-diol [nM]	−	11 (10, 12)	16 (14, 18)	32 (28, 36)
	+	8.1 (7.4, 8.9) *	11 (10, 12) *	22 (19, 24) *
		G(WP): F = 36.6, *p* < 0.001; S: F = 114, *p* < 0.001; G × S: F = 0, *p* = 0.972; GDM− b > a, c > a, c > b; GDM+ b > a, c > a, c > b		
5α-Androstane-3β,17β-diol [pM]	−	110 (92, 130)	110 (90, 130)	100 (87, 120)
	+	82 (69, 97)	87 (74, 100)	91 (77, 110)
		G(UP): F = 4.5, *p* = 0.036; S: F = 0, *p* = 0.975; G × S: F = 0.2, *p* = 0.792		
Conjugated 5α-androstane-3β,17β-diol [nM]	−	10 (9, 12)	13 (12, 15)	20 (17, 23)
	+	7.9 (7, 8.9) *	8.7 (7.7, 9.9) *	14 (13, 16) *
		G(WP): F = 20.8, *p* < 0.001; S: F = 25.1, *p* < 0.001; G × S: F = 0.4, *p* = 0.673; GDM− c > a, c > b; GDM+ c > a, c > b		
Conjugated 5β-androstane-3β,17β-diol [nM]	−	0.68 (0.56, 0.83)	0.84 (0.69, 1)	1.2 (1, 1.5)
	+	0.46 (0.38, 0.55) *	0.43 (0.36, 0.52) *	0.73 (0.6, 0.89) *
		G(WP): F = 24.3, *p* < 0.001; S: F = 10.1, *p* < 0.001; G × S: F = 0.6, *p* = 0.574; GDM− c > a, c > b; GDM+ c > a, c > b		
Cortisone [nM]	−	150 (140, 160)	180 (170, 200)	210 (200, 230)
	+	140 (130, 160)	170 (150, 180)	180 (160, 190) *
		G(UP): F = 6, *p* = 0.016; S: F = 15, *p* < 0.001; G × S: F = 1.1, *p* = 0.344; GDM− b > a, c > a; GDM+ c > a		
11-Deoxycorticosterone [nM]	−	0.64 (0.47, 0.86)	0.99 (0.75, 1.3)	1.2 (0.92, 1.6)
	+	0.3 (0.21, 0.41) *	0.82 (0.62, 1.1)	1.1 (0.8, 1.4)
		G(UP): F = 4.5, *p* = 0.037; S: F = 12.3, *p* < 0.001; G × S: F = 1.2, *p* = 0.315; GDM− c > a; GDM+ b > a, c > a		
Conjugated 11-deoxycorticosterone [nM]	−	5.2 (4.1, 6.5)	5 (4, 6.3)	6.4 (5.1, 8.2)
	+	3.5 (2.8, 4.3)	3.9 (3.1, 4.8)	4.8 (3.8, 6.1)
		G(UP): F = 5.9, *p* = 0.017; S: F = 1.8, *p* = 0.164; G × S: F = 0.1, *p* = 0.886		
3α,5α-Tetrahydrocorticosterone [pM]	−	38 (27, 54)	35 (25, 49)	150 (110, 220)
	+	55 (40, 77)	65 (47, 91)	190 (140, 280)
		G(UP): F = 4.2, *p* = 0.043; S: F = 20.7, *p* < 0.001; G × S: F = 0.4, *p* = 0.67; GDM− c > a, c > b; GDM+ c > a, c > b		
11β-Hydroxyandrosterone [nM]	−	0.5 (0.43, 0.57)	0.47 (0.41, 0.54)	0.94 (0.84, 1.1)
	+	0.31 (0.27, 0.35) *	0.27 (0.23, 0.3) *	0.64 (0.57, 0.73) *
		G(WP): F = 43.7, *p* < 0.001; S: F = 55.8, *p* < 0.001; G × S: F = 0.6, *p* = 0.581; GDM− c > a, c > b; GDM+ c > a, c > b		
11β-Hydroxyandrosterone sulfate [nM]	−	8.5 (7.8, 9.3)	9.7 (8.9, 11)	13 (12, 14)
	+	6.2 (5.7, 6.7) *	8 (7.4, 8.8) *	12 (11, 13)
		G(WP): F = 18.1, *p* < 0.001; S: F = 38.6, *p* < 0.001; G × S: F = 1.7, *p* = 0.195; GDM− c > a, c > b; GDM+ b > a, c > a, c > b		
11β-Hydroxyepiandrosterone [pM]	−	14 (11, 18)	18 (14, 23)	52 (41, 66)
	+	7.3 (5.5, 9.6) *	12 (8.9, 15)	37 (29, 46)
		G(UP): F = 10.5, *p* = 0.002; S: F = 41, *p* < 0.001; G × S: F = 0.3, *p* = 0.721; GDM− c > a, c > b; GDM+ c > a, c > b		

ANOVA model: G: … factor GDM (GDM+ vs. GDM−); S: … factor Stage, G × S: … GDM × Stage interaction, F = F-statistic, *p* = *p* value, * represents a significant difference between GDM+ and GDM− subgroups (*p* < 0.05), a, b, and c symbolize stages Week 24−28, Week 30−36, and Labor, respectively, only significant differences between stages are shown (*p* < 0.05), WP…well-powered analysis, UP…underpowered analysis.

**Table 2:** Profiles of product to precursor ratios that may reflect the balance between 17-hydroxy-steroids and corresponding 17-deoxy-steroids and possibly also the activity of CYP17A1 (17-hydroxylase step) in the GDM− and GDM+ groups from week 24 of gestation to labor.

Product to Precursor Ratio	GDM	GDM × Stage		
		Week 24–28 of Gestation (a)	Week 30–36 of Gestation (b)	At Labor (c)
17-Hydroxy-pregnenolone/pregnenolone	−	0.68 (0.59, 0.77)	0.76 (0.66, 0.87)	1.9 (1.6, 2.3)
	+	0.54 (0.47, 0.61)	0.64 (0.56, 0.73)	2.3 (1.9, 2.7)
		G: F = 1.6, *p* = 0.207; S: F = 87.9, *p* < 0.001; G × S: F = 2.1, *p* = 0.133; GDM− c > a, c > b; GDM+ c > a, c > b		
1000 × 17-Hydroxy-pregnenolone/pregnenolone, sulfates	−	54 (49, 60)	67 (60, 74)	93 (84, 100)
	+	55 (50, 61)	69 (63, 76)	92 (83, 100)
		G: F = 0.1, *p* = 0.81; S: F = 30.1, *p* < 0.001; G × S: F = 0, *p* = 0.96; GDM− b > a, c > a, c > b; GDM+ b > a, c > a, c > b		
1000 × 17-Hydroxy-progesterone/progesterone	−	39 (36, 43)	46 (42, 50)	76 (70, 83)
	+	37 (34, 40)	40 (37, 43)	67 (62, 73)
		G(UP): F = 6, *p* = 0.016; S: F = 71.5, *p* < 0.001; G × S: F = 0.2, *p* = 0.805; GDM− c > a, c > b; GDM+ c > a, c > b		
1000 × 17-Hydroxy-allopregnanolone/allopregnanolone	−	16 (14, 18)	20 (18, 22)	21 (18, 23)
	+	8.2 (7.1, 9.4) *	15 (13, 17) *	16 (14, 18) *
		G(WP): F = 31.3, *p* < 0.001; S: F = 17.4, *p* < 0.001; G × S: F = 2.5, *p* = 0.089; GDM− c > a; GDM+ b > a, c > a		
1000 × 17-Hydroxy-allopregnanolone/allopregnanolone, sulfates	−	15 (14, 16)	17 (16, 19)	21 (20, 23)
	+	14 (13, 15)	14 (13, 15) *	17 (16, 19) *
		G(WP): F = 15.3, *p* < 0.001; S: F = 14.9, *p* < 0.001; G × S: F = 1.4, *p* = 0.255; GDM− c > a, c > b; GDM+ c > a, c > b		
1000 × 17-Hydroxy-pregnanolone/pregnanolone	−	35 (33, 39)	44 (40, 48)	65 (59, 72)
	+	29 (27, 31) *	37 (34, 40) *	56 (51, 62)
		G(WP): F = 13.7, *p* < 0.001; S: F = 59.1, *p* < 0.001; G × S: F = 0.3, *p* = 0.75; GDM− b > a, c > a, c > b; GDM+ b > a, c > a, c > b		
1000 × 17-Hydroxy-pregnanolone/pregnanolone, conjugates	−	97 (90, 100)	120 (110, 130)	150 (140, 170)
	+	97 (91, 100)	92 (86, 98) *	130 (120, 140) *
		G(UP): F = 11, *p* = 0.001; S: F = 32.8, *p* < 0.001; G × S: F = 3.3, *p* = 0.04; GDM− b > a, c > a, c > b; GDM+ c > a, c > b		

The symbols are the same as for Table 1.

**Table 3:** Profiles of product to precursor ratios that may reflect balance between 17-hydroxy-steroids and corresponding C19-steroids and possibly also the activity of CYP17A1 (C17,20-lyase step) in the GDM− and GDM+ groups from week 24 of gestation to labor.

Product to Precursor Ratio	GDM	GDM × Stage		
		Week 24–28 of Gestation (a)	Week 30–36 of Gestation (b)	Labor (c)
DHEA/17-hydroxy-pregnenolone	−	1.1 (0.99, 1.1)	0.97 (0.91, 1)	0.9 (0.84, 0.97)
	+	1.1 (1.1, 1.2)	0.88 (0.82, 0.94)	0.65 (0.6, 0.7) *
		G(UP): F = 9.2, *p* = 0.003; S: F = 25.7, *p* < 0.001; G × S(WP): F = 7.9, *p* < 0.001; GDM− c < a; GDM+ b < a, c < a, c < b		
DHEA/17-hydroxy-pregnenolone, sulfates	−	130 (120, 150)	88 (76, 100)	45 (39, 52)
	+	150 (130, 170)	88 (77, 100)	49 (42, 56)
		G: F = 0.5, *p* = 0.475; S: F = 69.8, *p* < 0.001; G × S: F = 0.1, *p* = 0.874; GDM− b < a, c < a, c < b; GDM+ b < a, c < a, c < b		
1000 × Androstenedione/17-hydroxyprogesterone	−	0.43 (0.38, 0.48)	0.33 (0.29, 0.37)	0.35 (0.31, 0.4)
	+	0.4 (0.36, 0.45)	0.3 (0.26, 0.33)	0.44 (0.39, 0.49)
		G: F = 0.1, *p* = 0.737; S: F = 7.9, *p* < 0.001; G × S: F = 2.6, *p* = 0.082; GDM− b < a; GDM+ b < a, c > b		
1000 × Androsterone/17-hydroxyallopregnanolone	−	1.4 (1.2, 1.7)	1.1 (0.97, 1.3)	1.3 (1.1, 1.5)
	+	2.7 (2.3, 3.2) *	1.4 (1.2, 1.6)	1.4 (1.2, 1.6)
		G(UP): F = 12.3, *p* < 0.001; S: F = 11, *p* < 0.001; G × S: F = 2.8, *p* = 0.069; GDM−; GDM+ b < a, c < a		
Androsterone/17-hydroxy-allopregnanolone, sulfates	−	32 (28, 36)	21 (18, 24)	21 (18, 24)
	+	49 (44, 55) *	28 (25, 31) *	24 (21, 27)
		G(WP): F = 17.8, *p* < 0.001; S: F = 29.7, *p* < 0.001; G × S: F = 2, *p* = 0.147; GDM− b < a, c < a; GDM+ b < a, c < a		
1000 × Etiocholanolone/17-hydroxypregnanolone	−	0.28 (0.25, 0.32)	0.21 (0.19, 0.23)	0.23 (0.21, 0.26)
	+	0.34 (0.3, 0.38)	0.23 (0.21, 0.25)	0.26 (0.24, 0.29)
		G(UP): F = 4.5, *p* = 0.037; S: F = 12.3, *p* < 0.001; G × S: F = 0.2, *p* = 0.809; GDM− b < a; GDM+ b < a, c < a		
1000 × Etiocholanolone/17-hydroxypregnanolone, conjugates	−	0.64 (0.58, 0.7)	0.38 (0.34, 0.42)	0.37 (0.33, 0.41)
	+	0.82 (0.75, 0.9) *	0.46 (0.42, 0.51)	0.45 (0.4, 0.49)
		G(WP): F = 14.7, *p* < 0.001; S: F = 47, *p* < 0.001; G × S: F = 0.3, *p* = 0.78; GDM− b < a, c < a; GDM+ b < a, c < a		

The symbols are the same as for Table 1.

**Table 4:** Profiles of product to precursor ratios that may reflect the SRD5As activities in the GDM− and GDM+ groups from week 24 of gestation to labor.

Product to Precursor Ratio	GDM	GDM × Stage		
		Week 24–28 of Gestation (a)	Week 30–36 of Gestation (b)	Labor (c)
1000 × (5α-Dihydroprogesterone + allopregnanolone + isopregnanolone)/progesterone	−	0.25 (0.23, 0.27)	0.24 (0.22, 0.26)	0.3 (0.27, 0.33)
	+	0.21 (0.2, 0.23)	0.21 (0.19, 0.23)	0.3 (0.27, 0.33)
		G: F = 3.6, *p* = 0.06; S: F = 14.7, *p* < 0.001; G × S: F = 1.1, *p* = 0.335; GDM− c > b; GDM+ c > a, c > b		
(5α,20α-Tetrahydroprogesterone + 5α/β-Pregnane-3β,20α-diols)/20α-dihydroprogesterone	−	1.5 (1.4, 1.6)	1.3 (1.2, 1.4)	1.5 (1.4, 1.5)
	+	1.2 (1.1, 1.3) *	1.2 (1.2, 1.3)	1.4 (1.3, 1.5)
		G(UP): F = 8.9, *p* = 0.004; S: F = 4.7, *p* = 0.012; G × S: F = 2.5, *p* = 0.087; GDM− b < a; GDM+ c > a, c > b		
1000 × 17-Hydroxyallopregnanolone/17-hydroxyprogesterone	−	25 (22, 30)	23 (20, 26)	20 (17, 23)
	+	12 (9.8, 14) *	18 (15, 21)	18 (15, 21)
		G(WP): F = 17.5, *p* < 0.001; S: F = 1.1, *p* = 0.323; G × S(WP): F = 5.9, *p* = 0.004; GDM+ b > a, c > a		
1000 × 5α-Pregnane-3α/β,17,20α-triols/17,20α-dihydroxy-4-pregnene-3-one	−	68 (61, 76)	44 (40, 49)	35 (32, 39)
	+	55 (50, 61) *	40 (36, 44)	35 (32, 39)
		G: F = 3.5, *p* = 0.066; S: F = 36.3, *p* < 0.001; G × S: F = 1.2, *p* = 0.31; GDM− b < a, c < a, c < b; GDM+ b < a, c < a		
(5α-Androstane-3,17-dione + androsterone + epiandrosterone)/androstenedione	−	0.22 (0.2, 0.23)	0.18 (0.17, 0.19)	0.19 (0.18, 0.2)
	+	0.2 (0.18, 0.21)	0.17 (0.16, 0.18)	0.17 (0.16, 0.18)
		G: F = 3.9, *p* = 0.051; S: F = 7.3, *p* = 0.001; G × S: F = 0.5, *p* = 0.631; GDM− b < a; GDM+ c < a		
(5α-Dihydrotestosterone + 5α-androstane-3α/β,17β-diols)/testosterone	−	0.29 (0.25, 0.33)	0.24 (0.21, 0.27)	0.18 (0.16, 0.21)
	+	0.26 (0.22, 0.3)	0.21 (0.18, 0.25)	0.18 (0.16, 0.21)
		G: F = 1, *p* = 0.325; S: F = 8.6, *p* < 0.001; G × S: F = 0.2, *p* = 0.846; GDM− c < a; GDM+ c < a		
1000 × (11β-Hydroxyandrosterone + 11β-hydroxyepiandrosterone)/11β-hydroxyandrostenedione	−	7.3 (6.5, 8.3)	7.1 (6.3, 8.1)	11 (9.8, 12)
	+	4.3 (3.8, 4.8) *	4.3 (3.8, 4.8) *	6.1 (5.4, 6.8) *
		G(WP): F = 64, *p* < 0.001; S: F = 16.7, *p* < 0.001; G × S: F = 0.2, *p* = 0.828; GDM− c > a, c > b; GDM+ c > a, c > b		

The symbols are the same as for Table 1.

**Table 5:** Profiles of product to precursor ratios that may reflect the balance between 17β-hydroxy- and 17-oxo-steroids in the GDM− and GDM+ groups from week 24 of gestation to labor.

Product to Precursor Ratios	GDM	GDM × Stage		
		Week 24–28 of Gestation (a)	Week 30–36 of Gestation (b)	Labor (c)
Androstenediol/DHEA	−	0.21 (0.2, 0.23)	0.19 (0.18, 0.21)	0.2 (0.18, 0.21)
	+	0.19 (0.18, 0.2)	0.19 (0.18, 0.2)	0.16 (0.15, 0.17) *
		G(UP): F = 7.5, *p* = 0.007; S: F = 3.5, *p* = 0.035; G × S: F = 1.9, *p* = 0.154; GDM+ c < a, c < b		
5-Androstene-3β,7α,17β-triol/7α-hydroxy-DHEA	−	0.4 (0.35, 0.46)	0.31 (0.28, 0.36)	0.33 (0.29, 0.38)
	+	0.56 (0.49, 0.66) *	0.42 (0.37, 0.48) *	0.33 (0.29, 0.38)
		G(UP): F = 7.7, *p* = 0.007; S: F = 7.7, *p* < 0.001; G × S: F = 2.1, *p* = 0.128; GDM+ b < a, c < a		
5-Androstene-3β,7β,17β-triol/7β-hydroxy-DHEA	−	0.1 (0.087, 0.12)	0.14 (0.12, 0.16)	0.25 (0.21, 0.29)
	+	0.16 (0.14, 0.19) *	0.22 (0.19, 0.26) *	0.31 (0.26, 0.36)
		G(WP): F = 16.3, *p* < 0.001; S: F = 23.6, *p* < 0.001; G × S: F = 0.7, *p* = 0.502; GDM− c > a, c > b; GDM+ c > a, c > b		
Testosterone/androstenedione	−	0.43 (0.4, 0.46)	0.37 (0.34, 0.4)	0.38 (0.36, 0.41)
	+	0.35 (0.32, 0.37) *	0.35 (0.32, 0.38)	0.32 (0.3, 0.35) *
		G(UP): F = 11.9, *p* < 0.001; S: F = 1.9, *p* = 0.161; G × S: F = 1.5, *p* = 0.239; GDM− b < a		
Estradiol/estrone	−	3.9 (3.4, 4.6)	4.3 (3.7, 5)	2.5 (2.1, 2.8)
	+	3.4 (2.9, 3.9)	3.8 (3.3, 4.4)	1.9 (1.7, 2.2)
		G(UP): F = 4.8, *p* = 0.031; S: F = 22.7, *p* < 0.001; G × S: F = 0.2, *p* = 0.805; GDM− c < a, c < b; GDM+ c < a, c < b		
5α-Dihydrotestosterone/5α-androstane-3,17-dione	−	0.77 (0.68, 0.86)	0.71 (0.62, 0.8)	0.47 (0.41, 0.54)
	+	0.74 (0.64, 0.85)	0.62 (0.53, 0.71)	0.59 (0.5, 0.69)
		G: F = 0, *p* = 0.898; S: F = 7, *p* = 0.002; G × S: F = 1.7, *p* = 0.192; GDM− c < a, c < b		
5α-Androstane-3α,17β-diol/androsterone	−	0.25 (0.23, 0.28)	0.26 (0.23, 0.28)	0.22 (0.2, 0.24)
	+	0.27 (0.25, 0.3)	0.25 (0.22, 0.27)	0.21 (0.19, 0.23)
		G: F = 0, *p* = 0.995; S: F = 5.7, *p* = 0.005; G × S: F = 0.6, *p* = 0.558; GDM+ c < a		
5α-Androstane-3β,17β-diol/epiandrosterone	−	0.5 (0.4, 0.62)	0.51 (0.42, 0.64)	0.23 (0.18, 0.29)
	+	0.59 (0.48, 0.73)	0.47 (0.37, 0.58)	0.28 (0.22, 0.35)
		G: F = 0.5, *p* = 0.494; S: F = 15.6, *p* < 0.001; G × S: F = 0.6, *p* = 0.553; GDM− c < a, c < b; GDM+ c < a, c < b		
1000 × 11β-Hydroxy-testosterone/11β-hydroxyandrostenedione	−	71 (49, 100)	100 (74, 140)	110 (78, 150)
	+	61 (38, 91)	100 (70, 140)	62 (39, 93)
		G: F = 1.3, *p* = 0.255; S: F = 1.6, *p* = 0.214; G × S: F = 0.7, *p* = 0.521		

The symbols are the same as for Table 1.

**Table 6:** Profiles of sulfated to unsulfated steroid ratios in the GDM− and GDM+ groups from week 24 of gestation to labor.

Steroid	GDM	GDM × Stage		
		Week 24–28 of Gestation (a)	Week 30–36 of Gestation (b)	Labor (c)
Pregnenolone	−	39 (35, 43)	37 (34, 41)	82 (72, 94)
	+	26 (23, 28) *	28 (26, 31) *	78 (69, 89)
		G(WP): F = 20.8, *p* < 0.001; S: F = 106.1, *p* < 0.001; G × S(WP): F = 4.3, *p* = 0.017; GDM− c > a, c > b; GDM+ c > a, c > b		
20α-Dihydropregnenolone	−	230 (210, 250)	230 (210, 250)	170 (160, 190)
	+	210 (190, 230)	240 (220, 260)	220 (200, 240) *
		G: F = 1.3, *p* = 0.267; S: F = 5.9, *p* = 0.004; G × S(WP): F = 3.7, *p* = 0.028; GDM− c < a, c < b		
17-Hydroxypregnenolone	−	3 (2.7, 3.3)	3.2 (2.9, 3.5)	3 (2.7, 3.3)
	+	2.6 (2.4, 2.9)	2.9 (2.6, 3.1)	3.2 (2.9, 3.5)
		G: F = 1, *p* = 0.316; S: F = 1, *p* = 0.366; G × S: F = 1.2, *p* = 0.31		
16α-Hydroxypregnenolone	−	74 (57, 97)	80 (61, 100)	79 (61, 100)
	+	55 (42, 72)	49 (37, 65)	72 (55, 95)
		G: F = 3.6, *p* = 0.06; S: F = 0.6, *p* = 0.528; G × S: F = 0.6, *p* = 0.568		
DHEA	−	350 (320, 390)	270 (240, 300)	170 (150, 190)
	+	320 (290, 360)	290 (260, 320)	180 (160, 200)
		G: F = 0, *p* = 0.837; S: F = 39.4, *p* < 0.001; G × S: F = 0.7, *p* = 0.507; GDM− b < a, c < a, c < b; GDM+ c < a, c < b		
Androstenediol	−	140 (120, 170)	170 (140, 200)	140 (120, 170)
	+	150 (130, 180)	180 (160, 220)	230 (190, 270) *
		G(UP): F = 4.5, *p* = 0.037; S: F = 2.2, *p* = 0.115; G × S: F = 2.2, *p* = 0.12; GDM+ c > a		
5-Androstene-3β,16α,17β-triol	−	160 (140, 180)	170 (150, 190)	160 (150, 180)
	+	140 (120, 160)	150 (130, 170)	190 (170, 220)
		G: F = 0.3, *p* = 0.575; S: F = 2.4, *p* = 0.1; G × S: F = 2.4, *p* = 0.094; GDM+ c > a, c > b		
Estrone	−	5.9 (4.4, 8.1)	7.1 (5.2, 9.7)	7.5 (5.6, 10)
	+	4.9 (3.6, 6.5)	5.4 (4.1, 7.3)	5.2 (3.9, 7)
		G: F = 2.7, *p* = 0.106; S: F = 0.4, *p* = 0.697; G × S: F = 0.1, *p* = 0.918		
Estradiol	−	0.54 (0.43, 0.67)	0.59 (0.47, 0.74)	0.75 (0.61, 0.94)
	+	0.5 (0.4, 0.62)	0.53 (0.43, 0.66)	0.61 (0.49, 0.76)
		G: F = 1.1, *p* = 0.293; S: F = 1.7, *p* = 0.186; G × S: F = 0.1, *p* = 0.885		
Estriol	−	4.5 (3.6, 5.6)	4.9 (3.9, 6)	7.1 (5.8, 8.8)
	+	3.8 (3.1, 4.6)	3.3 (2.7, 4)	6.5 (5.3, 8.1)
		G: F = 3.4, *p* = 0.068; S: F = 8.9, *p* < 0.001; G × S: F = 0.6, *p* = 0.533; GDM− c > a; GDM+ c > a, c > b		
Allopregnanolone	−	39 (36, 42)	42 (38, 45)	45 (41, 49)
	+	26 (24, 29) *	36 (33, 39)	45 (42, 49)
		G(WP): F = 13.6, *p* < 0.001; S: F = 17.9, *p* < 0.001; G × S(WP): F = 6.2, *p* = 0.003; GDM+ b > a, c > a, c > b		
Isopregnanolone	−	68 (63, 74)	74 (68, 81)	110 (99, 120)
	+	50 (46, 54) *	63 (59, 69)	120 (110, 130)
		G(UP): F = 6.3, *p* = 0.014; S: F = 77.9, *p* < 0.001; G × S(WP): F = 8.3, *p* < 0.001; GDM− c > a, c > b; GDM+ b > a, c > a, c > b		
17-Hydroxyallopregnanolone	−	41 (36, 47)	45 (39, 52)	54 (47, 63)
	+	51 (44, 59)	37 (33, 43)	56 (48, 66)
		G: F = 0.1, *p* = 0.801; S: F = 4.9, *p* = 0.009; G × S: F = 2.2, *p* = 0.12; GDM+ b < a, c > b		
Androsterone	−	930 (850, 1000)	780 (710, 850)	800 (730, 870)
	+	970 (890, 1100)	890 (810, 970)	970 (890, 1100) *
		G(UP): F = 6.3, *p* = 0.014; S: F = 2.6, *p* = 0.077; G × S: F = 0.8, *p* = 0.439; GDM− b < a		
Epiandrosterone	−	590 (520, 680)	730 (630, 840)	370 (320, 420)
	+	460 (400, 530)	470 (410, 530) *	290 (250, 340)
		G(WP): F = 16.2, *p* < 0.001; S: F = 21.9, *p* < 0.001; G × S: F = 0.8, *p* = 0.476; GDM− c < a, c < b; GDM+ c < a, c < b		
11β-Hydroxyandrosterone	−	21 (19, 25)	28 (25, 33)	17 (15, 20)
	+	22 (20, 26)	33 (29, 38)	22 (19, 25)
		G: F = 3.3, *p* = 0.072; S: F = 13.2, *p* < 0.001; G × S: F = 0.5, *p* = 0.614; GDM− b > a, c < b; GDM+ b > a, c < b		
11β-Hydroxyepiandrosterone	−	280 (210, 380)	340 (250, 460)	160 (120, 210)
	+	450 (330, 630)	480 (350, 650)	210 (160, 270)
		G(UP): F = 4.4, *p* = 0.038; S: F = 9.8, *p* < 0.001; G × S: F = 0.1, *p* = 0.881; GDM− c < a, c < b; GDM+ c < a, c < b		

The symbols are the same as for Table 1.

**Table 7:** Profiles of product to precursor ratios that may reflect HSD3Bs activities in the GDM− and GDM+ groups from week 24 of gestation to labor.

Product to Precursor Ratios	GDM	GDM × Stage		
		Week 24–28 of Gestation (a)	Week 30–36 of Gestation (b)	At Labor (c)
Progesterone/pregnenolone	−	70 (62, 78)	90 (80, 100)	91 (80, 100)
	+	62 (55, 70)	80 (71, 90)	65 (58, 73) *
		G(UP): F = 7.7, *p* = 0.006; S: F = 5.1, *p* = 0.008; G × S: F = 1.1, *p* = 0.333; GDM− b > a, c > a; GDM+ b > a		
20α-Dihydroprogesterone/20α-dihydropregnenolone	−	36 (33, 40)	63 (58, 69)	45 (41, 49)
	+	38 (36, 42)	58 (54, 62)	43 (40, 47)
		G: F = 0.3, *p* = 0.599; S: F = 44.8, *p* < 0.001; G × S: F = 1.1, *p* = 0.35; GDM− b > a, c > a, c < b; GDM+ b > a, c < b		
17-Hydroxyprogesterone/17-hydroxypregnenolone	−	4.1 (3.5, 4.8)	5.6 (4.8, 6.5)	2.8 (2.3, 3.4)
	+	4.5 (3.8, 5.2)	5.3 (4.5, 6.1)	2.1 (1.7, 2.5)
		G: F = 0.8, *p* = 0.374; S: F = 24.3, *p* < 0.001; G × S: F = 1.2, *p* = 0.314; GDM− c < a, c < b; GDM+ c < a, c < b		
16α-Hydroxyprogesterone/16α-hydroxypregnenolone	−	19 (17, 21)	22 (20, 25)	18 (16, 20)
	+	23 (21, 25)	25 (23, 28)	18 (16, 20)
		G(UP): F = 4.4, *p* = 0.038; S: F = 7.5, *p* < 0.001; G × S: F = 0.8, *p* = 0.477; GDM− c < b; GDM+ c < a, c < b		
Androstenedione/DHEA	−	1.5 (1.4, 1.7)	1.7 (1.5, 1.8)	1.1 (1, 1.2)
	+	1.5 (1.3, 1.6)	1.7 (1.5, 1.8)	0.99 (0.91, 1.1)
		G: F = 1.3, *p* = 0.257; S: F = 32.4, *p* < 0.001; G × S: F = 0.7, *p* = 0.519; GDM− c < a, c < b; GDM+ c < a, c < b		
Testosterone/androstenediol	−	3 (2.8, 3.3)	3.2 (2.9, 3.5)	2.2 (2.1, 2.5)
	+	2.8 (2.6, 3.1)	3.1 (2.8, 3.4)	1.9 (1.7, 2) *
		G: F = 3.6, *p* = 0.062; S: F = 27.7, *p* < 0.001; G × S: F = 1, *p* = 0.385; GDM− c < a, c < b; GDM+ c < a, c < b		
16α-Hydroxytestosterone/5-androstene-3β,16α,17β-triol	−	7.4 (6.3, 8.7)	9.8 (8.4, 11)	5.4 (4.5, 6.5)
	+	6.1 (5.1, 7.2)	7.1 (6, 8.2) *	4.8 (3.9, 5.7)
		G(UP): F = 5.5, *p* = 0.021; S: F = 9.2, *p* < 0.001; G × S: F = 0.6, *p* = 0.58; GDM− c < b; GDM+ c < b		

The symbols are the same as for Table 1.

**Table 8:** Profiles of product to precursor ratios that may reflect the conversion of adrenal androgens to their 7α/β-hydroxy-derivatives in the GDM− and GDM+ groups from week 24 of gestation to labor.

Product to Precursor Ratios	GDM	GDM × Stage		
		Week 24–28 of Gestation (a)	Week 30–36 of Gestation (b)	At Labor (c)
1000 × 7α-Hydroxy-DHEA/DHEA	-	82 (75, 91)	86 (78, 94)	94 (86, 100)
	+	62 (56, 69) *	84 (77, 92)	78 (71, 86) *
		G(UP): F = 9.1, *p* = 0.003; S: F = 4.6, *p* = 0.012; G × S: F = 1.9, *p* = 0.153; GDM+ b > a, c > a		
1000 × 5-Androstene-3β,7α,17β-triol/androstenediol	-	150 (140, 170)	150 (140, 170)	170 (160, 190)
	+	190 (170, 210)	190 (170, 210)	150 (140, 170)
		G: F = 2.5, *p* = 0.114; S: F = 0.2, *p* = 0.798; G × S: F = 3.6, *p* = 0.031; GDM+ c < a		
1000 × 7β-Hydroxy-DHEA/DHEA	-	86 (74, 98)	81 (70, 93)	58 (49, 68)
	+	53 (44, 62) *	71 (61, 81)	48 (41, 57)
		G(UP): F = 9.5, *p* = 0.003; S: F = 6.1, *p* = 0.003; G × S: F = 1.7, *p* = 0.183; GDM− c < a, c < b; GDM+ c < b		
1000 × 5-Androstene-3β,7α,17β-triol/androstenediol	-	44 (36, 53)	60 (50, 72)	85 (72, 100)
	+	51 (42, 62)	69 (58, 82)	75 (63, 88)
		G: F = 0.2, *p* = 0.629; S: F = 9.5, *p* < 0.001; G × S: F = 0.9, *p* = 0.412; GDM− c > a, c > b; GDM+ c > a		

The symbols are the same as for Table 1.

**Table 9:** Profiles of product to precursor ratios that may reflect HSD11B1 activity in the GDM− and GDM+ groups from week 24 of gestation to labor.

Product to Precursor Ratios	GDM	GDM × Stage		
		Week 24–28 of Gestation (a)	Week 30–36 of Gestation (b)	At Labor (c)
7-oxo-DHEA/7α-hydroxy-DHEA	−	1.3 (1.1, 1.5)	2 (1.6, 2.3)	1.1 (0.94, 1.4)
	+	1.6 (1.4, 2)	1.9 (1.6, 2.2)	1.1 (0.94, 1.4)
		G: F = 0.5, *p* = 0.488; S: F = 9, *p* < 0.001; G × S: F = 0.8, *p* = 0.459; GDM− b > a, c < b; GDM+ c < b		
5-Androstene-3β,7β,17β-triol/5-androstene-3β,7α,17β-triol	−	0.27 (0.23, 0.32)	0.34 (0.29, 0.39)	0.44 (0.38, 0.51)
	+	0.29 (0.25, 0.34)	0.36 (0.31, 0.42)	0.46 (0.4, 0.53)
		G: F = 0.6, *p* = 0.428; S: F = 10.6, *p* < 0.001; G × S: F = 0, *p* = 0.975; GDM− c > a; GDM+ c > a		
7β-Hydroxy-DHEA/7-oxo-DHEA	−	0.97 (0.82, 1.1)	0.61 (0.52, 0.71)	0.54 (0.46, 0.64)
	+	0.5 (0.43, 0.59) *	0.42 (0.36, 0.48) *	0.5 (0.43, 0.58)
		G(WP): F = 17, *p* < 0.001; S: F = 5.7, *p* = 0.005; G × S: F = 3.5, *p* = 0.035; GDM− b < a, c < a		
7β-Hydroxy-DHEA/7α-hydroxy-DHEA	−	1 (0.91, 1.1)	0.9 (0.79, 1)	0.6 (0.51, 0.7)
	+	0.8 (0.69, 0.91)	0.82 (0.71, 0.92)	0.65 (0.56, 0.74)
		G: F = 2, *p* = 0.166; S: F = 9.4, *p* < 0.001; G × S: F = 1.7, *p* = 0.195; GDM− c < a, c < b		
Cortisol/cortisone	−	4.2 (3.9, 4.5)	3.5 (3.3, 3.8)	4.8 (4.4, 5.1)
	+	4.5 (4.2, 4.9)	4.1 (3.9, 4.4) *	6 (5.5, 6.5) *
		G(WP): F = 14.1, *p* < 0.001; S: F = 22.6, *p* < 0.001; G × S: F = 0.9, *p* = 0.414; GDM− b < a, c > b; GDM+ c > a, c > b		

The symbols are the same as for Table 1.

## Data Availability

Not applicable.

## Supplementary Materials

The following are available online at https://www.mdpi.com/article/10.3390/biom11121746/s1, Table S1: Profiles of steroids in the GDM− and GDM+ groups from week 24 of gestation to labor unrelated to GDM, Table S2: Levels of steroids in mixed umbilical cord blood of GDM− and GDM+ women at labor, Table S3: Profiles of product to precursor ratios that may reflect the AKR1D1 activity in the GDM− and GDM+ groups from week 24 of gestation to labor, Table S4: Profiles of product to precursor ratios that may reflect the activity of 16α-hydroxylating enzymes in the GDM− and GDM+ groups from week 24 of gestation to labor, Table S5: Profiles of product to precursor ratios that may reflect the activity of CYP19A1 (aromatase) in the GDM− and GDM+ groups from week 24 of gestation to labor.
