# Supplementary material for: Altered Steroidome in Women with Gestational Diabetes Mellitus: Focus on Neuroactive and Immunomodulatory Steroids from the 24th Week of Pregnancy to Labor
Source: Biomolecules. 2021 Nov 23;11(12):1746. doi: 10.3390/biom11121746 (PMC8698588; doi:10.3390/biom11121746)
Supplement: Supplementary file 1 [file biomolecules-11-01746-s001.zip › Table S1 steroids unrelated to GDM.pdf]

Supplementary Table S1. Profiles of steroids in the GDM- and GDM+ groups from week 24 of gestation to labor unrelated to GDM

Steroid	GDM	GDM × Stage<br>Week 24–28 of gestation (a)	Week 30–36 of gestation (b)	Labor (c)
Pregnenolone sulfate [nM]	−	170 (160, 180)	180 (160, 190)	330 (300, 360)
	+	150 (140, 160)	170 (160, 180)	360 (330, 400)
		G: F=0.9, p=0.354; S: F=133.7, p<0.001; G×S: F=2.4, p=0.096; GDM− c>a, c>b; GDM+ c>a, c>b		
17-Hydroxypregnenolone [nM]	−	2.9 (2.5, 3.4)	3.6 (3.1, 4.2)	8.1 (6.7, 9.9)
	+	3 (2.6, 3.5)	3.8 (3.3, 4.4)	9.4 (7.7, 12)
		G: F=0.6, p=0.447; S: F=51.8, p<0.001; G×S: F=0.1, p=0.93; GDM− c>a, c>b; GDM+ c>a, c>b		
17-Hydroxypregnenolone sulfate [nM]	−	9.6 (8.4, 11)	12 (11, 14)	33 (28, 39)
	+	8.7 (7.6, 9.9)	12 (11, 14)	39 (33, 46)
		G: F=0, p=0.962; S: F=112.3, p<0.001; G×S: F=1, p=0.391; GDM− c>a, c>b; GDM+ b>a, c>a, c>b		
16α-Hydroxypregnenolone [nM]	−	0.51 (0.45, 0.57)	0.89 (0.79, 0.99)	1.4 (1.2, 1.5)
	+	0.49 (0.44, 0.55)	0.89 (0.8, 1)	1.3 (1.2, 1.4)
		G: F=0.2, p=0.63; S: F=80.9, p<0.001; G×S: F=0.1, p=0.893; GDM− b>a, c>a, c>b; GDM+ b>a, c>a, c>b		
20α-Dihydropregnenolone [nM]	−	1.2 (1.1, 1.3)	1.3 (1.2, 1.4)	2.5 (2.3, 2.7)
	+	1.4 (1.3, 1.5)	1.4 (1.3, 1.5)	2.2 (2, 2.4)
		G: F=0.4, p=0.544; S: F=76.3, p<0.001; G×S: F=2.6, p=0.079; GDM− c>a, c>b; GDM+ c>a, c>b		
Dehydroepiandrosterone (DHEA) [nM]	−	3.3 (2.9, 3.7)	3.8 (3.3, 4.3)	8.4 (7.5, 9.6)
	+	3.5 (3, 3.9)	3.5 (3.1, 4)	8.5 (7.5, 9.6)
		G: F=0, p=0.886; S: F=72, p<0.001; G×S: F=0.3, p=0.729; GDM− c>a, c>b; GDM+ c>a, c>b		
7-oxo-DHEA [nM]	−	0.39 (0.3, 0.48)	0.63 (0.52, 0.75)	1 (0.87, 1.2)
	+	0.47 (0.38, 0.57)	0.63 (0.53, 0.75)	0.95 (0.82, 1.1)
		G: F=0.1, p=0.749; S: F=24.8, p<0.001; G×S: F=0.5, p=0.614; GDM− b>a, c>a, c>b; GDM+ c>a, c>b		
5-Androstene-3β,17β-diol (androstenediol) [nM]	−	0.67 (0.59, 0.76)	0.74 (0.65, 0.83)	1.5 (1.3, 1.7)
	+	0.63 (0.56, 0.71)	0.63 (0.56, 0.71)	1.4 (1.2, 1.6)
		G: F=1.9, p=0.172; S: F=47.7, p<0.001; G×S: F=0.2, p=0.85; GDM− c>a, c>b; GDM+ c>a, c>b,		
Androstenediol sulfate [nM]	−	120 (100, 130)	140 (120, 160)	270 (240, 300)
	+	120 (110, 140)	150 (140, 170)	310 (280, 350)
		G: F=1.9, p=0.171; S: F=63.5, p<0.001; G×S: F=0.3, p=0.711; GDM− c>a, c>b; GDM+ c>a, c>b		
5-Androstene-3β,7β,17β-triol [pM]	−	34 (28, 42)	45 (37, 54)	130 (110, 160)
	+	30 (24, 37)	45 (37, 54)	110 (90, 120)
		G: F=1.6, p=0.212; S: F=64.4, p<0.001; G×S: F=0.6, p=0.572; GDM− c>a, c>b; GDM+ c>a, c>b		
17-Hydroxyprogesterone [nM]	−	11 (10, 13)	18 (16, 21)	24 (22, 27)
	+	13 (12, 15)	20 (17, 22)	20 (18, 23)
		G: F=0, p=0.85; S: F=34.3, p<0.001; G×S: F=2.2, p=0.117; GDM− b>a, c>a, c>b; GDM+ b>a, c>a		
17α,20α-Dihydroxy-4-pregnene-3-one [nM]	−	3.7 (3.2, 4.3)	6.5 (5.7, 7.6)	10 (9, 12)
	+	3.5 (3, 4)	6.3 (5.5, 7.2)	8.8 (7.6, 10)
		G: F=1.2, p=0.27; S: F=52.2, p<0.001; G×S: F=0.2, p=0.808; GDM− b>a, c>a, c>b; GDM+ b>a, c>a, c>b		
16α-Hydroxyprogesterone [nM]	−	8.6 (7.5, 9.9)	18 (16, 21)	29 (25, 34)
	+	11 (9.2, 12)	22 (19, 25)	20 (17, 22)*
		G: F=0, p=0.914; S: F=54.4, p<0.001; G×S: F=6.3, p=0.003; GDM− b>a, c>a, c>b; GDM+ b>a, c>a		
Conjugated 20α-dihydroprogesterone [nM]	−	14 (12, 15)	23 (21, 25)	32 (29, 36)
	+	15 (14, 17)	23 (21, 26)	32 (29, 35)
		G: F=0.4, p=0.539; S: F=71.1, p<0.001; G×S: F=0.3, p=0.72; GDM− b>a, c>a, c>b; GDM+ b>a, c>a, c>b		
Androstenedione [nM]	−	5.2 (4.8, 5.7)	6.3 (5.8, 6.9)	8.9 (8.1, 9.9)
	+	5.2 (4.8, 5.7)	6 (5.5, 6.6)	8.9 (8.1, 9.9)
		G: F=0.1, p=0.743; S: F=37.1, p<0.001; G×S: F=0.1, p=0.89; GDM− b>a, c>a, c>b; GDM+ c>a, c>b		
Conjugated testosterone [nM]	−	4.7 (4, 5.6)	8.1 (6.8, 9.5)	14 (12, 16)
	+	4.3 (3.6, 5.1)	6.8 (5.8, 8)	14 (12, 16)
		G: F=1, p=0.318; S: F=51.7, p<0.001; G×S: F=0.2, p=0.804; GDM− b>a, c>a, c>b; GDM+ b>a, c>a, c>b		
Conjugated epitestosterone [nM]	−	11 (10, 13)	17 (16, 19)	21 (19, 24)
	+	11 (9.6, 12)	16 (15, 18)	25 (23, 28)
		G: F=0.1, p=0.74; S: F=67.9, p<0.001; G×S: F=2, p=0.138; GDM− b>a, c>a, c>b; GDM+ b>a, c>a, c>b		
5α-Dihydrotestosterone [nM]	−	0.32 (0.28, 0.37)	0.31 (0.27, 0.36)	0.33 (0.29, 0.38)
	+	0.33 (0.28, 0.39)	0.27 (0.23, 0.32)	0.37 (0.31, 0.43)
		G: F=0, p=0.94; S: F=1.8, p=0.179; G×S: F=0.7, p=0.487		

Supplementary Table S1. Profiles of steroids in the GDM- and GDM+ groups from week 24 of gestation to labor unrelated to GDM

Steroid	GDM	GDM × Stage		
		Week 24–28 of gestation (a)	Week 30–36 of gestation (b)	Labor (c)
Conjugated 5α-dihydrotestosterone [nM]	–	1.1 (0.92, 1.4)	1.2 (0.97, 1.4)	2 (1.7, 2.3)
	+	1 (0.81, 1.2)	1.2 (1, 1.5)	2.5 (2.1, 2.9)
		G: F=0.4, p=0.546; S: F=20.8, p<0.001; G×S: F=1.1, p=0.33; GDM− c>a, c>b; GDM+ c>a, c>b		
Estrone [nM]	–	17 (14, 21)	19 (15, 23)	27 (22, 33)
	+	15 (12, 18)	19 (16, 23)	25 (20, 30)
		G: F=0.5, p=0.48; S: F=6.5, p=0.002; G×S: F=0.2, p=0.824; GDM− c>a; GDM+ c>a		
Estriol [nM]	–	41 (33, 50)	61 (50, 74)	52 (43, 63)
	+	32 (26, 40)	62 (52, 75)	47 (39, 57)
		G: F=0.8, p=0.387; S: F=7.9, p<0.001; G×S: F=0.4, p=0.676; GDM− b>a; GDM+ b>a		
5α-Dihydroprogesterone [nM]	–	50 (45, 56)	69 (62, 76)	65 (58, 72)
	+	49 (44, 54)	67 (61, 75)	61 (54, 68)
		G: F=0.3, p=0.575; S: F=10.1, p<0.001; G×S: F=0.1, p=0.95; GDM− b>a, c>a; GDM+ b>a		
Allopregnanolone [nM]	–	22 (20, 23)	28 (26, 30)	27 (25, 29)
	+	21 (20, 23)	28 (26, 30)	25 (23, 27)
		G: F=0.4, p=0.54; S: F=12.5, p<0.001; G×S: F=0.3, p=0.769; GDM− b>a, c>a; GDM+ b>a, c>a		
Isopregnanolone [nM]	–	6.9 (6.2, 7.7)	9.4 (8.5, 10)	6.9 (6.2, 7.7)
	+	6.8 (6.1, 7.5)	9 (8.2, 10)	6.4 (5.7, 7.1)
		G: F=0.6, p=0.431; S: F=13.3, p<0.001; G×S: F=0.1, p=0.908; GDM− b>a, c<b; GDM+ b>a, c<b		
5β-Dihydroprogesterone [nM]	–	1.3 (0.99, 1.6)	1.1 (0.86, 1.4)	0.42 (0.31, 0.55)
	+	1.1 (0.89, 1.4)	1.2 (0.92, 1.4)	0.41 (0.3, 0.54)
		G: F=0, p=0.878; S: F=23.5, p<0.001; G×S: F=0.1, p=0.866; GDM− c<a, c<b; GDM+ c<a, c<b		
Pregnanolone [nM]	–	22 (20, 24)	22 (20, 24)	17 (16, 19)
	+	22 (20, 24)	23 (21, 25)	16 (14, 17)
		G: F=0, p=0.851; S: F=14.3, p<0.001; G×S: F=0.8, p=0.453; GDM− c<a, c<b; GDM+ c<a, c<b		
Epipregnanolone [nM]	–	1.5 (1.3, 1.6)	1.5 (1.3, 1.6)	1 (0.89, 1.1)
	+	1.4 (1.3, 1.6)	1.3 (1.1, 1.4)	0.95 (0.84, 1.1)
		G: F=1.4, p=0.235; S: F=14.5, p<0.001; G×S: F=0.3, p=0.717; GDM− c<a, c<b; GDM+ c<a, c<b		
5α,20α-Tetrahydroprogesterone [nM]	–	17 (15, 18)	27 (25, 30)	34 (31, 38)
	+	16 (15, 18)	25 (22, 27)	29 (26, 32)
		G: F=3.3, p=0.073; S: F=53, p<0.001; G×S: F=0.7, p=0.505; GDM− b>a, c>a, c>b; GDM+ b>a, c>a		
Conjugated 5α,20α-tetrahydroprogesterone [nM]	–	17 (15, 20)	28 (24, 33)	58 (48, 73)
	+	19 (16, 22)	30 (26, 36)	53 (44, 65)
		G: F=0.2, p=0.644; S: F=48.8, p<0.001; G×S: F=0.5, p=0.615; GDM− b>a, c>a, c>b; GDM+ b>a, c>a, c>b		
5α-Pregnane-3α,20α-diol [nM]	–	25 (23, 27)	45 (41, 50)	63 (57, 69)
	+	26 (24, 28)	40 (37, 43)	51 (47, 56)*
		G: F=3.2, p=0.076; S: F=92.1, p<0.001; G×S: F=2.2, p=0.117; GDM− b>a, c>a, c>b; GDM+ b>a, c>a, c>b		
5α-Pregnane-3β,20α-diol [nM]	–	19 (17, 21)	30 (27, 33)	48 (44, 52)
	+	18 (16, 20)	30 (27, 32)	41 (38, 45)
		G: F=1.5, p=0.22; S: F=84.9, p<0.001; G×S: F=0.7, p=0.517; GDM− b>a, c>a, c>b; GDM+ b>a, c>a, c>b		
5β,20α-Tetrahydroprogesterone [nM]	–	1.8 (1.6, 2)	1.8 (1.6, 2)	1.3 (1.2, 1.5)
	+	1.7 (1.6, 1.9)	1.9 (1.8, 2.1)	1.2 (1.1, 1.3)
		G: F=0, p=0.843; S: F=15.9, p<0.001; G×S: F=0.8, p=0.437; GDM− c<a, c<b; GDM+ c<a, c<b		
Conjugated 5β,20α-tetrahydroprogesterone [nM]	–	10 (9, 12)	12 (10, 14)	18 (16, 22)
	+	11 (9.6, 13)	15 (12, 17)	22 (18, 27)
		G: F=2.8, p=0.096; S: F=16.3, p<0.001; G×S: F=0.2, p=0.795; GDM− c>a, c>b; GDM+ c>a, c>b		
Conjugated 5β-pregnane-3α,20α-diol [μM]	–	1 (0.91, 1.1)	1.2 (1.1, 1.3)	1.4 (1.3, 1.5)
	+	0.99 (0.91, 1.1)	1.2 (1.1, 1.3)	1.4 (1.3, 1.6)
		G: F=0.1, p=0.753; S: F=16.6, p<0.001; G×S: F=0.1, p=0.936; GDM− b>a, c>a; GDM+ b>a, c>a		
5β-Pregnane-3β,20α-diol [nM]	–	0.56 (0.47, 0.68)	0.71 (0.59, 0.85)	0.69 (0.57, 0.83)
	+	0.67 (0.55, 0.81)	0.53 (0.44, 0.63)	0.53 (0.44, 0.63)
		G: F=1.6, p=0.208; S: F=0, p=0.988; G×S: F=2.2, p=0.119; GDM+		
Conjugated 5β-pregnane-3β,20α-diol [μM]	–	0.8 (0.73, 0.88)	0.92 (0.84, 1)	1.1 (0.97, 1.2)
	+	0.75 (0.68, 0.83)	0.92 (0.84, 1)	1.1 (1, 1.2)
		G: F=0, p=0.845; S: F=13, p<0.001; G×S: F=0.3, p=0.745; GDM− c>a; GDM+ b>a, c>a		
	–	120 (100, 150)	150 (120, 180)	200 (170, 250)

Supplementary Table S1. Profiles of steroids in the GDM- and GDM+ groups from week 24 of gestation to labor unrelated to GDM

Steroid	GDM	GDM × Stage Week 24–28 of gestation (a)	Week 30–36 of gestation (b)	Labor (c)
Conjugated 5α-pregnane-3α,17α,20α-triol [nM]	+	130 (110, 150)	170 (140, 200)	170 (140, 210)
		G: F=0, p=0.957; S: F=6, p=0.003; G×S: F=0.7, p=0.507; GDM− c>a; GDM+		
5α-Androstane-3,17-dione [nM]	−	0.42 (0.38, 0.46)	0.42 (0.38, 0.46)	0.67 (0.6, 0.75)
	+	0.4 (0.36, 0.44)	0.39 (0.36, 0.44)	0.56 (0.51, 0.63)
		G: F=2.4, p=0.123; S: F=23.6, p<0.001; G×S: F=0.5, p=0.621; GDM− c>a, c>b; GDM+ c>a, c>b		
Androsterone sulfate [nM]	−	470 (440, 510)	440 (410, 480)	570 (530, 620)
	+	470 (430, 510)	440 (400, 480)	580 (530, 630)
		G: F=0, p=0.954; S: F=13.3, p<0.001; G×S: F=0, p=0.991; GDM− c>a, c>b; GDM+ c>a, c>b		
Epiandrosterone [nM]	−	0.18 (0.15, 0.21)	0.13 (0.12, 0.16)	0.34 (0.29, 0.38)
	+	0.15 (0.13, 0.18)	0.14 (0.12, 0.16)	0.32 (0.28, 0.37)
		G: F=0.5, p=0.504; S: F=45.6, p<0.001; G×S: F=0.5, p=0.612; GDM− c>a, c>b; GDM+ c>a, c>b		
Etiocholanolone [nM]	−	0.22 (0.2, 0.24)	0.2 (0.19, 0.22)	0.27 (0.25, 0.29)
	+	0.24 (0.22, 0.26)	0.22 (0.21, 0.24)	0.27 (0.25, 0.29)
		G: F=2.1, p=0.15; S: F=10.1, p<0.001; G×S: F=0.5, p=0.64; GDM− c>a, c>b; GDM+ c>b		
Epietiocholanolone sulfate [nM]	−	6.7 (5.8, 7.8)	6.6 (5.8, 7.7)	7.8 (6.7, 8.9)
	+	7.4 (6.4, 8.5)	6.3 (5.5, 7.3)	8 (6.9, 9.2)
		G: F=0.1, p=0.763; S: F=2.1, p=0.131; G×S: F=0.3, p=0.745; GDM+		
Conjugated 5β-androstane-3α,17β-diol [nM]	−	2.6 (2.3, 2.8)	3.3 (3, 3.6)	5.2 (4.7, 5.8)
	+	2.7 (2.4, 2.9)	3.4 (3.1, 3.8)	5.7 (5.2, 6.3)
		G: F=1.1, p=0.289; S: F=69.6, p<0.001; G×S: F=0.1, p=0.929; GDM− b>a, c>a, c>b; GDM+ b>a, c>a, c>b		
Cortisol [nM]	−	620 (590, 660)	650 (620, 680)	1000 (980, 1100)
	+	620 (590, 650)	670 (640, 700)	1100 (1000, 1200)
		G: F=0.5, p=0.485; S: F=129.2, p<0.001; G×S: F=0.2, p=0.799; GDM− c>a, c>b; GDM+ c>a, c>b		
21-Deoxycortisol [nM]	−	0.23 (0.17, 0.32)	0.24 (0.18, 0.32)	0.53 (0.4, 0.71)
	+	0.27 (0.2, 0.36)	0.25 (0.19, 0.34)	0.44 (0.33, 0.59)
		G: F=0, p=0.961; S: F=7.9, p<0.001; G×S: F=0.4, p=0.676; GDM− c>a, c>b; GDM+		
11-Deoxycortisol [nM]	−	4 (2.9, 5.5)	5.9 (4.3, 7.9)	8.5 (6.3, 12)
	+	3.5 (2.4, 5)	6.2 (4.3, 8.7)	8.3 (5.9, 12)
		G: F=0, p=0.849; S: F=6.6, p=0.003; G×S: F=0.1, p=0.92; GDM− c>a; GDM+ c>a		
3α,5β-Tetrahydrocorticosterone [pM]	−	160 (110, 210)	140 (100, 190)	570 (430, 770)
	+	98 (73, 130)	150 (110, 200)	440 (330, 580)
		G: F=1.7, p=0.191; S: F=30.8, p<0.001; G×S: F=0.9, p=0.418; GDM− c>a, c>b; GDM+ c>a, c>b		
11β-Hydroxyandrostenedione [nM]	−	66 (60, 72)	70 (64, 76)	99 (90, 110)
	+	72 (66, 79)	74 (67, 80)	110 (100, 120)
		G: F=2.7, p=0.102; S: F=27, p<0.001; G×S: F=0.1, p=0.905; GDM− c>a, c>b; GDM+ c>a, c>b		
11β-Hydroxytestosterone [nM]	−	5 (3.4, 7)	7.7 (5.6, 10)	9.5 (7.1, 12)
	+	5.2 (3.4, 7.6)	8.3 (5.8, 11)	7 (4.7, 9.7)
		G: F=0.2, p=0.701; S: F=2.5, p=0.087; G×S: F=0.5, p=0.622; GDM− c>a		
11β-Hydroxyepiandrosterone sulfate [nM]	−	3.6 (3.1, 4)	5.1 (4.5, 5.8)	6.4 (5.6, 7.2)
	+	3.2 (2.8, 3.6)	5.3 (4.7, 6)	7.1 (6.3, 8)
		G: F=0.1, p=0.816; S: F=35.6, p<0.001; G×S: F=0.9, p=0.429; GDM− b>a, c>a; GDM+ b>a, c>a, c>b		
11β-Hydroxyetiocholanolone [nM]	−	0.45 (0.38, 0.54)	0.44 (0.36, 0.52)	0.8 (0.68, 0.93)
	+	0.58 (0.49, 0.68)	0.45 (0.38, 0.53)	0.89 (0.76, 1)
		G: F=2, p=0.158; S: F=18.8, p<0.001; G×S: F=0.5, p=0.641; GDM− c>a, c>b; GDM+ c>a, c>b		
11β-Hydroxyetiocholanolone sulfate [nM]	−	2.3 (2.1, 2.7)	2.7 (2.4, 3.1)	3 (2.7, 3.4)
	+	2.5 (2.2, 2.8)	2.9 (2.6, 3.2)	3.5 (3.1, 3.9)
		G: F=1.6, p=0.205; S: F=6.2, p=0.003; G×S: F=0.2, p=0.817; GDM− c>a; GDM+ c>a		

*ANOVA model: G: … factor GDM (GDM+ vs. GDM−); S: … factor Stage, G×S: … GDM × Stage interaction, F = F-statistic, p = p-value, *represents a significant difference between GDM+ and GDM− subgroups (p<0.05), a, b, and c symbolize stages Week 24–28, Week 30–36, and Labor, respectively, only significant differences between stages are shown (p<0.05)*

52 steroidů
