# Supplementary material for: Altered Steroidome in Women with Gestational Diabetes Mellitus: Focus on Neuroactive and Immunomodulatory Steroids from the 24th Week of Pregnancy to Labor
Source: Biomolecules. 2021 Nov 23;11(12):1746. doi: 10.3390/biom11121746 (PMC8698588; doi:10.3390/biom11121746)
Supplement: Supplementary file 1 [file biomolecules-11-01746-s001.zip › Table S2, Steroids, cord blood.pdf]

Supplementary Table S2. Levels of steroids in mixed umbilical cord blood of GDM- and GDM+ women at labor

No	Steroids	GDM−	GDM+	p-value
1	Pregnenolone [nM]	48 (38, 60)	46 (38, 57)	0.875
2	Pregnenolone sulfate [μM]	2.8 (2.3, 3.4)	2.9 (2.4, 3.4)	0.885
3	17-Hydroxypregnenolone [nM]	29 (23, 36)	22 (18, 26)	0.115
4	17-Hydroxypregnenolone sulfate [μM]	7.7 (6.2, 9.3)	5.6 (4.5, 6.9)	0.087
5	16α-Hydroxypregnenolone [nM]	11 (9.7, 13)	9.9 (8.5, 11)	0.310
6	16α-Hydroxypregnenolone sulfate [μM]	4.6 (2.6, 8.3)	2.7 (1.6, 4.4)	0.243
7	20α-Dihydropregnenolone [nM]	3 (2.5, 3.6)	2.4 (2.1, 2.8)	0.162
8	20α-Dihydropregnenolone sulfate [μM]	1.1 (0.9, 1.3)	1.3 (1.1, 1.6)	0.238
9	Dehydroepiandrosterone (DHEA) [nM]	4.3 (3.5, 5.2)	3.2 (2.6, 3.8)	0.092
10	DHEA sulfate [μM]	4.7 (3.7, 5.9)	3.4 (2.6, 4.2)	0.082
11	7α-Hydroxy-DHEA [nM]	1.2 (0.91, 1.5)	1 (0.78, 1.3)	0.441
12	7-oxo-DHEA [nM]	1.4 (1.1, 1.8)	1.2 (0.96, 1.5)	0.467
13	7β-Hydroxy-DHEA [nM]	0.3 (0.2, 0.43)	0.19 (0.13, 0.29)	0.238
14	Androstenediol, 5-Androstene-3β, 17β-diol [nM]	0.47 (0.42, 0.54)	0.46 (0.41, 0.52)	0.875
15	Androstenediol sulfate [μM]	11 (9.1, 14)	9.8 (7.8, 12)	0.395
16	5-Androstene-3β,7α,17β-triol [pM]	51 (35, 72)	46 (33, 64)	0.749
17	5-Androstene-3β,7β,17β-triol [pM]	50 (33, 73)	25 (16, 38)	0.060
18	5-Androstene-3β,16α,17β-triol [nM]	8 (6.7, 9.7)	6.4 (5.4, 7.5)	0.126
19	5-Androstene-3β,16α,17β-triol sulfate [μM]	0.86 (0.69, 1.1)	0.74 (0.6, 0.9)	0.399
20	Progesterone [μM]	2.1 (1.7, 2.6)	2.6 (2.2, 3.1)	0.225
21	17-Hydroxyprogesterone [nM]	93 (80, 110)	92 (80, 110)	0.975
22	17α,20α-Dihydroxy-4-pregnene-3-one [nM]	35 (26, 47)	30 (23, 39)	0.544
23	Conjugated 17α,20α-dihydroxy-4-pregnen-3-one [nM]	24 (16, 35)	22 (16, 31)	0.804
24	16α-Hydroxyprogesterone [nM]	190 (150, 240)	210 (170, 260)	0.577
25	20α-Dihydroprogesterone [nM]	140 (110, 190)	130 (100, 170)	0.628
26	Conjugated 20α-dihydroprogesterone [nM]	84 (66, 110)	85 (68, 110)	0.936
27	Androstenedione [nM]	4.3 (3.6, 5.4)	4.3 (3.6, 5.2)	0.927
28	Testosterone [nM]	0.58 (0.43, 0.76)	0.38 (0.28, 0.5)	0.092
29	Conjugated testosterone [nM]	54 (44, 66)	46 (38, 56)	0.330
30	16α-Hydroxytestosterone [nM]	21 (17, 27)	17 (14, 21)	0.243
31	Conjugated epitestosterone [nM]	500 (430, 580)	490 (430, 560)	0.860
32	5α-Dihydrotestosterone [pM]	92 (69, 120)	91 (67, 120)	0.986
33	Conjugated 5α-dihydrotestosterone [nM]	0.93 (0.6, 1.4)	0.94 (0.63, 1.4)	0.977
34	Estrone [nM]	48 (36, 63)	51 (39, 66)	0.771
35	Estrone sulfate [nM]	35 (25, 49)	31 (23, 42)	0.644
36	Estradiol [nM]	35 (26, 47)	27 (20, 35)	0.261
37	Estradiol sulfate [nM]	20 (15, 26)	18 (14, 22)	0.569
38	Estriol [μM]	0.66 (0.52, 0.86)	0.63 (0.5, 0.81)	0.817
39	Estriol sulfate [μM]	2.9 (2.2, 4)	2.1 (1.6, 2.7)	0.148
40	5α-Dihydroprogesterone [nM]	150 (130, 180)	150 (130, 180)	0.952
41	Allopregnanolone [nM]	25 (21, 30)	22 (19, 26)	0.413
42	Allopregnanolone sulfate [nM]	340 (280, 420)	280 (230, 330)	0.182
43	Isopregnanolone [nM]	32 (26, 39)	29 (25, 35)	0.615
44	Isopregnanolone sulfate [nM]	320 (280, 370)	260 (230, 300)	0.083
45	5β-Dihydroprogesterone [nM]	9.7 (6.4, 14)	16 (12, 21)	0.095
46	Pregnanolone [nM]	21 (18, 24)	20 (17, 23)	0.682
47	Conjugated pregnanolone [nM]	300 (250, 370)	270 (220, 320)	0.417
48	Epipregnanolone [nM]	2.5 (2.1, 3)	2.3 (1.9, 2.7)	0.525
49	Conjugated epipregnanolone [nM]	94 (78, 110)	74 (63, 87)	0.100
50	5α,20α-Tetrahydroprogesterone [nM]	46 (39, 56)	44 (37, 52)	0.738
51	Conjugated 5α,20α-tetrahydroprogesterone [nM]	100 (77, 130)	81 (64, 100)	0.322
52	5α-Pregnane-3α,20α-diol [nM]	13 (10, 16)	13 (10, 16)	0.897
53	Conjugated 5α-pregnane-3α,20α-diol [μM]	4.2 (3.5, 5.2)	3.9 (3.3, 4.7)	0.634
54	5α-Pregnane-3β,20α-diol [nM]	55 (45, 69)	50 (42, 61)	0.580
55	Conjugated 5α-pregnane-3β,20α-diol [μM]	13 (10, 15)	11 (9.3, 13)	0.435
56	5β,20α-Tetrahydroprogesterone [nM]	12 (9.5, 15)	11 (8.4, 14)	0.553
57	Conjugated 5β,20α-tetrahydroprogesterone [nM]	49 (38, 63)	51 (40, 63)	0.881
58	5β-Pregnane-3α,20α-diol [nM]	6.2 (5.1, 7.8)	5.9 (4.8, 7.2)	0.726
59	Conjugated 5β-pregnane-3α,20α-diol [μM]	0.92 (0.75, 1.1)	0.87 (0.72, 1)	0.714

60	5β-Pregnane-3β,20α-diol [nM]	1.5 (1.2, 1.9)	1.2 (0.95, 1.5)	0.258
61	Conjugated 5β-pregnane-3β,20α-diol [nM]	490 (420, 580)	450 (390, 530)	0.567
62	17-Hydroxyallopregnanolone [nM]	0.95 (0.76, 1.2)	0.58 (0.45, 0.74)	0.020
63	17-Hydroxyallopregnanolone sulfate [nM]	11 (9.1, 13)	8 (6.8, 9.4)	0.035
64	17-Hydroxypregnanolone [nM]	1.2 (0.98, 1.4)	0.89 (0.76, 1)	0.062
65	Conjugated 17-hydroxypregnanolone [nM]	80 (67, 93)	57 (48, 67)	0.021
66	5α-Pregnane-3α,17,20α-triol [nM]	0.2 (0.14, 0.28)	0.12 (0.085, 0.18)	0.145
67	Conjugated 5α-pregnane-3α,17α,20α-triol [nM]	98 (72, 130)	100 (78, 140)	0.840
68	5α-Pregnane-3β,17,20α-triol [nM]	0.098 (0.074, 0.13)	0.065 (0.05, 0.085)	0.098
69	Conjugated 5α-pregnane-3β,17α,20α-triol [nM]	4.4 (3, 6.5)	3.7 (2.6, 5.2)	0.554
70	5β-Pregnane-3α,17,20α-triol [nM]	2.2 (1.7, 2.7)	1.6 (1.3, 2)	0.111
71	Conjugated 5β-opregnane-3α,17α,20α-triol [nM]	400 (290, 550)	450 (340, 600)	0.667
72	5α-Androstane-3,17-dione [nM]	0.33 (0.29, 0.39)	0.36 (0.31, 0.41)	0.582
73	Androsterone [nM]	0.18 (0.15, 0.22)	0.14 (0.11, 0.17)	0.098
74	Androsterone sulfate [nM]	75 (59, 94)	64 (53, 79)	0.438
75	Epiandrosterone [nM]	0.16 (0.13, 0.2)	0.12 (0.095, 0.15)	0.142
76	Epiandrosterone sulfate [nM]	56 (48, 65)	43 (36, 50)	0.056
77	Etiocholanolone [nM]	0.16 (0.13, 0.21)	0.13 (0.1, 0.16)	0.174
78	Etiocholanolone sulfate [nM]	12 (9.9, 15)	11 (8.7, 13)	0.377
79	Epietiocholanolone sulfate [nM]	0.98 (0.7, 1.4)	0.81 (0.61, 1.1)	0.499
80	5α-Androstane-3α,17β-diol [nM]	0.07 (0.039, 0.12)	0.055 (0.032, 0.092)	0.612
81	Conjugated 5α-androstane-3α,17β-diol [nM]	22 (18, 26)	15 (13, 18)	0.034
82	5α-Androstane-3β,17β-diol [nM]	0.052 (0.025, 0.1)	0.056 (0.028, 0.11)	0.900
83	Conjugated 5α-androstane-3β,17β-diol [nM]	11 (8.8, 15)	7.7 (6.1, 9.6)	0.059
84	Conjugated 5β-androstane-3α,17β-diol [nM]	6.5 (5.6, 7.5)	5.7 (5, 6.6)	0.335
85	Conjugated 5β-androstane-3β,17β-diol [nM]	0.36 (0.25, 0.53)	0.3 (0.21, 0.42)	0.527
86	Cortisol [nM]	120 (94, 160)	130 (100, 160)	0.838
87	Cortisone [nM]	280 (230, 340)	310 (260, 370)	0.587
88	Corticosterone [nM]	21 (16, 27)	19 (15, 24)	0.652
89	21-Deoxycortisol [nM]	0.4 (0.25, 0.67)	0.33 (0.22, 0.52)	0.625
90	11-Deoxycortisol [nM]	40 (29, 57)	30 (22, 42)	0.315
91	11-Deoxycorticosterone [nM]	4.6 (3.6, 5.7)	5.9 (4.8, 7.1)	0.171
92	Conjugated 11-deoxycorticosterone [nM]	75 (45, 130)	88 (54, 150)	0.716
93	3α,5α-Tetrahydrocorticosterone [nM]	0.23 (0.13, 0.42)	0.12 (0.072, 0.2)	0.158
94	3α,5β-Tetrahydrocorticosterone [nM]	0.41 (0.23, 0.74)	0.18 (0.11, 0.3)	0.094
95	11β-Hydroxyandrostenedione [nM]	20 (15, 25)	18 (14, 22)	0.643
96	11β-Hydroxytestosterone [nM]	19 (12, 29)	15 (9.2, 23)	0.523
97	11β-Hydroxyandrosterone [nM]	0.27 (0.18, 0.4)	0.15 (0.1, 0.21)	0.075
98	11β-Hydroxyandrosterone sulfate [nM]	8 (6.2, 10)	6.6 (5.2, 8.3)	0.337
99	11β-Hydroxyepiandrosterone [nM]	0.087 (0.061, 0.12)	0.071 (0.05, 0.099)	0.489
100	11β-Hydroxyepiandrosterone sulfate [nM]	8.6 (6.4, 11)	8.9 (6.8, 11)	0.892
101	11β-Hydroxyetiocholanolone [nM]	0.43 (0.29, 0.63)	0.41 (0.28, 0.59)	0.879
102	11β-Hydroxyetiocholanolone sulfate [nM]	3.4 (2.7, 4.1)	4.1 (3.4, 4.9)	0.259
