# Supplementary material for: Altered Steroidome in Women with Gestational Diabetes Mellitus: Focus on Neuroactive and Immunomodulatory Steroids from the 24th Week of Pregnancy to Labor
Source: Biomolecules. 2021 Nov 23;11(12):1746. doi: 10.3390/biom11121746 (PMC8698588; doi:10.3390/biom11121746)
Supplement: Supplementary file 1 [file biomolecules-11-01746-s001.zip › Table S3, CYP19A1.pdf]

Supplementary Table S3. Profiles of product to precursor ratios reflecting the activity of CYP19A1 (aromatase) in the GDM- and GDM+ groups from week 24 of gestation to labor

Product to precursor ratio	GDM	GDM × Stage<br>Week 24-28 of gestation (a)	Week 30-36 of gestation (b)	At labor (c)
Estrone/androstenedione	−	3.2 (2.5, 4.1)	3.2 (2.5, 4)	2.9 (2.3, 3.6)
	+	2.7 (2.1, 3.4)	3.1 (2.5, 3.8)	2.9 (2.3, 3.7)
		G: F=0.3, p=0.599; S: F=0.1, p=0.882; G×S: F=0.2, p=0.818		
Estradiol/testosterone	−	30 (25, 37)	37 (30, 45)	19 (16, 23)
	+	26 (21, 32)	41 (34, 50)	15 (12, 19)
		G: F=0.7, p=0.419; S: F=18.7, p<0.001; G×S: F=1, p=0.375; GDM− c<a, c<b; GDM+ b>a, c<a, c<b,		
