# Supplementary material for: Altered Steroidome in Women with Gestational Diabetes Mellitus: Focus on Neuroactive and Immunomodulatory Steroids from the 24th Week of Pregnancy to Labor
Source: Biomolecules. 2021 Nov 23;11(12):1746. doi: 10.3390/biom11121746 (PMC8698588; doi:10.3390/biom11121746)
Supplement: Supplementary file 1 [file biomolecules-11-01746-s001.zip › Table S4, CYP3A7.pdf]

Supplementary Table S4. Profiles of product to precursor ratios that may reflect the activity of 16α-hydroxylating enzymes in the GDM- and GDM+ groups from week 24 of gestation to labor

Product to precursor ratios	GDM	GDM × Stage<br>Week 24-28 of gestation (a)	 Week 30-36 of gestation (b)	 Labor (c)
16α-Hydroxy-pregnenolone/pregnenolone	−	0.12 (0.1, 0.13)	0.18 (0.16, 0.21)	0.34 (0.3, 0.38)
	+	0.087 (0.076, 0.1)*	0.15 (0.13, 0.17)	0.27 (0.24, 0.31)
		G: F=10.5, p=0.002; S: F=80.4, p<0.001; G×S: F=0.1, p=0.88; GDM− b>a, c>a, c>b; GDM+ b>a, c>a, c>b		
16α-Hydroxy-pregnenolone/pregnenolone, sulfates	−	0.27 (0.2, 0.36)	0.48 (0.36, 0.63)	0.4 (0.3, 0.54)
	+	0.2 (0.15, 0.27)	0.35 (0.26, 0.46)	0.34 (0.25, 0.45)
		G: F=2.6, p=0.107; S: F=4.7, p=0.011; G×S: F=0.1, p=0.942		
1000 × 16α-Hydroxy-progesterone/progesterone	−	31 (28, 33)	44 (40, 48)	72 (65, 79)
	+	30 (27, 32)	44 (40, 48)	63 (58, 70)
		G: F=0.9, p=0.358; S: F=90.8, p<0.001; G×S: F=0.5, p=0.621; GDM− b>a, c>a, c>b; GDM+ b>a, c>a, c>b		
5-Androstene-3β,16α,17β-triol/androstenediol	−	0.89 (0.78, 1)	1.3 (1.1, 1.4)	1.1 (0.95, 1.2)
	+	0.85 (0.74, 0.97)	1.3 (1.1, 1.5)	0.87 (0.76, 1)
		G: F=1.2, p=0.282; S: F=9.6, p<0.001; G×S: F=0.9, p=0.431; GDM− b>a; GDM+ b>a, c<b		
5-Androstene-3β,16α,17β-triol/androstenediol, sulfates	−	1.1 (0.96, 1.2)	1.4 (1.2, 1.6)	1.1 (1, 1.3)
	+	0.85 (0.75, 0.95)*	1.1 (0.95, 1.2)*	0.91 (0.81, 1)*
		G: F=14.5, p<0.001; S: F=5.3, p=0.007; G×S: F=0, p=0.963; GDM− b>a		
16α-Hydroxytestosterone/ testosterone	−	2 (1.6, 2.5)	3.4 (2.8, 4.1)	2.7 (2.2, 3.3)
	+	1.6 (1.3, 2)	3.2 (2.7, 3.9)	2.1 (1.6, 2.6)
		G: F=2.3, p=0.133; S: F=9.9, p<0.001; G×S: F=0.3, p=0.72; GDM− b>a; GDM+ b>a, c<b		
Estriol/estradiol	−	0.58 (0.53, 0.65)	0.66 (0.59, 0.73)	0.73 (0.66, 0.82)
	+	0.63 (0.57, 0.7)	0.82 (0.74, 0.91)*	0.82 (0.73, 0.92)
		G: F=5.2, p=0.025; S: F=6.5, p=0.002; G×S: F=0.5, p=0.585; GDM− c>a; GDM+ b>a, c>a		
Estriol/estradiol, sulfates	−	5.4 (4.7, 6.1)	5.7 (5.1, 6.5)	7.2 (6.3, 8.1)
	+	4.6 (4.1, 5.2)	5.6 (5, 6.3)	8.8 (7.8, 10)
		G: F=0, p=0.833; S: F=17.1, p<0.001; G×S: F=2.3, p=0.102; GDM− c>a; GDM+ c>a, c>b		

The symbols are the same as for Table 1
