# Supplementary material for: Altered Steroidome in Women with Gestational Diabetes Mellitus: Focus on Neuroactive and Immunomodulatory Steroids from the 24th Week of Pregnancy to Labor
Source: Biomolecules. 2021 Nov 23;11(12):1746. doi: 10.3390/biom11121746 (PMC8698588; doi:10.3390/biom11121746)
Supplement: Supplementary file 1 [file biomolecules-11-01746-s001.zip › Table S5, AKRD1.pdf]

Table S5. Profiles of product to precursor ratios that may reflect the AKR1D1 activity in the GDM- and GDM+ groups from week 24 of gestation to labor

		GDM × Stage		
Product to precursor ratios	GDM	Week 24-28 of gestation (a)	Week 30-36 of gestation (b)	Labor (c)
1000 × (5β-Dihydro-progesterone + pregnanolone + epipregnanolone)/ progesterone	−	9.5 (8.3, 11)	6.5 (5.6, 7.5)	5.6 (4.8, 6.4)
	+	8 (6.9, 9.1)	6.1 (5.3, 7)	4.7 (4.1, 5.5)
		G: F=2.9, p=0.089; S: F=15.4, p<0.001; G×S: F=0.2, p=0.829; GDM− b<a, c<a; GDM+ c<a		
1000 × (5β,20α-Tetrahydro-progesterone + 5β-pregnane-30α/β,20α-diols)/20α-dihydroprogesterone	−	63 (56, 70)	39 (35, 44)	24 (22, 27)
	+	55 (49, 62)	37 (33, 41)	21 (18, 23)
		G: F=3.2, p=0.075; S: F=78.9, p<0.001; G×S: F=0.2, p=0.803; GDM− b<a, c<a, c<b; GDM+ b<a, c<a, c<b		
1000 × 17-Hydroxypregnanolone/ 17-hydroxyprogesterone	−	65 (58, 72)	49 (44, 54)	46 (41, 51)
	+	47 (43, 52)*	44 (40, 49)	45 (40, 50)
		G: F=6.1, p=0.015; S: F=5, p=0.009; G×S: F=2.3, p=0.101; GDM− b<a, c<a		
5β-Pregnane-3α/β,17,20α-triols/17,20α-dihydroxy-4-pregnene-3-one	−	0.74 (0.68, 0.81)	0.45 (0.41, 0.49)	0.36 (0.33, 0.4)
	+	0.66 (0.61, 0.71)	0.5 (0.46, 0.54)	0.43 (0.39, 0.46)
		G: F=0.7, p=0.413; S: F=51.7, p<0.001; G×S: F=3.1, p=0.047; GDM− b<a, c<a, c<b; GDM+ b<a, c<a		
1000 × Etiocholanolone/ androstenedione	−	45 (42, 49)	34 (31, 37)	29 (26, 31)
	+	44 (40, 47)	36 (34, 39)	30 (28, 33)
		G: F=0.5, p=0.491; S: F=26.7, p<0.001; G×S: F=0.6, p=0.56; GDM− b<a, c<a, c<b; GDM+ b<a, c<a, c<b		
1000 × (11β-Hydroxyandrosterone + 11β-hydroxyepiandrosterone)/11β-hydroxyandrostenedione	−	7.3 (6.4, 8.2)	6.4 (5.6, 7.2)	7.4 (6.5, 8.4)
	+	7.8 (6.9, 8.7)	6 (5.3, 6.8)	8.6 (7.7, 9.6)
		G: F=0.7, p=0.419; S: F=5.1, p=0.008; G×S: F=0.8, p=0.459; GDM+ b<a, c>b		

The symbols are the same as for Table 1
